# Keratin-14 (KRT14) Positive Leader Cells Mediate Mesothelial Clearance and Invasion by Ovarian Cancer Cells

**DOI:** 10.3390/cancers11091228

**Published:** 2019-08-22

**Authors:** Maree Bilandzic, Adam Rainczuk, Emma Green, Nicole Fairweather, Thomas W. Jobling, Magdalena Plebanski, Andrew N. Stephens

**Affiliations:** 1 Hudson Institute of Medical Research, Clayton 3168, Australia; 2 Department of Molecular and Translational Sciences, Monash University, Clayton 3168, Australia; 3 Bruker Biosciences Pty Ltd., Preston 3078, Australia; 4 Department of Gynaecology Oncology Monash Health, Monash Medical Centre, Moorabbin 3189, Australia; 5 School of Health and Biomedical Sciences, RMIT University, Bundoora 3083, Australia

**Keywords:** cancer metastasis, leader cells, ovarian cancer, keratin 14, invasion

## Abstract

Epithelial ovarian cancer metastasis is driven by spheroids, which are heterogeneous cancer cell aggregates released from the primary tumour mass that passively disseminate throughout the peritoneal cavity to promote tumour spread, disease recurrence, and acquired chemoresistance. Despite their clinical importance, the molecular events that control spheroid attachment and invasion into underlying healthy tissues remain poorly understood. We examined a novel in vitro invasion model using imaging mass spectrometry to establish a “snapshot” of the spheroid/mesothelial interface. Amongst numerous adhesion-related proteins, we identified a sub-population of highly motile, invasive cells that expressed the basal epithelial marker KRT14 as an absolute determinant of invasive potential. The loss of KRT14 completely abrogated the invasive capacity, but had no impact on cell viability or proliferation, suggesting an invasion-specific role. Our data demonstrate KRT14 cells as an ovarian cancer “leader cell” phenotype underlying tumor invasion, and suggest their importance as a clinically relevant target in directed anti-tumour therapies.

## 1. Introduction

Ovarian cancer (OC) is the most lethal of the gynecological malignancies, with a ~70% five-year mortality [1]. Characterised by asymptomatic progression, patients typically present with advanced, extensively disseminated cancer, multiple tumor deposits, and ascitic fluid in the peritoneal cavity. Surgical debulking and platinum-based chemotherapy are standard of care, with most patients responding well to initial treatment. However, in over 90% of cases, women will develop recurrent, platinum-resistant disease, leaving limited therapeutic options underlying the high mortality rate.

Unlike the majority of epithelial tumor types, the primary mode for metastatic OC spread involves the exfoliation of heterogeneous, multicellular aggregates from the primary tumor site. Spheroids passively disseminate throughout the peritoneal cavity; on reaching a permissive environment, they attach and superficially invade through the mesothelial lining and implant onto adjacent organs [1,2,3,4]. Spheroids also persist in the peritoneal cavity following therapy, suggesting their potential involvement in tumor recurrence. Whilst emerging evidence suggests that OC cells may additionally disseminate via haematogenous and/or lymphatogenous routes [5], they rarely form metastatic deposits outside the peritoneum; the molecular basis underlying this preferential colonisation remains unknown [4].

Recent in vitro analyses have provided the first direct evidence highlighting how OC tumor deposition may occur, with integrin engagement in motile cells leading to the significant restructuring of extracellular matrix (ECM) components at the mesothelium. The generation of a contractile force that is sufficient enough to disrupt the mesothelial layer depends on the coordinated function of integrins, talins, and myosin II, to generate a conduit for invasive cells to infiltrate the underlying matrix [6].

In several solid tumour types, it has been demonstrated that only a subset of cancer cells mediate invasion. These cancer “leader cells” (LCs) maintain cell–cell adhesions with trailing epithelial cells to promote the deposition of multicellular aggregates of cells [1]. Whilst LCs have been demonstrated in multiple tumour types (breast, bladder, prostate, others), their presence and identity in OC remains undefined.

We have utilised a novel in vitro model, recapitulating key aspects of the peritoneal microenvironment [7] to examine the earliest protein-mediated events in epithelial ovarian cancer (EOC) attachment and invasion through a mesothelial monolayer. Using a combination of imaging mass spectrometry (IMS), CRISPR-mediated gene knockout and immunohistochemistry, we identified and validated several cancer-specific candidate proteins with potential roles in the metastatic spread of disease. In particular, the expression of the basal epithelial marker keratin-14 (KRT14) was identified as a key regulator of spheroid integrity, mesothelial attachment, and invasion into a sub-mesothelial matrix. Our data strongly suggest that, similar to other epithelial cancer types, KRT14 marks the LC sub-population in OCs and plays a significant role in mediating the metastatic spread of disease.

## 2. Results

### 2.1. Modelling the Invasive Interface In Vitro

**Figure 1:**
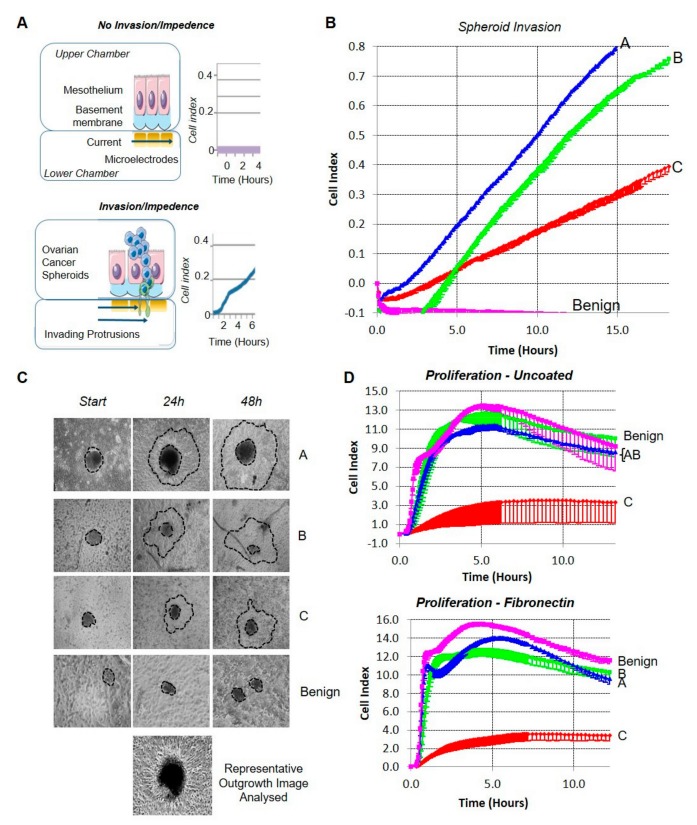
(**A**) Peritoneal microenvironment model. A mesothelial monolayer is grown on the basement membrane in the upper chamber of an xCELLigence CIM plate; the lower chamber contains media and microelectrodes. Disruption of the mesothelial cell layer (clearance) and the invasion of ovarian cancer cells alters the current flow and creates impedance, resulting in an increased cell index as a measure of invasion. (**B**) Real-time cell analysis (RTCA) invasion assay. Three patient-derived high-grade serous (HGS) ovarian cancer/mesothelial co-cultures (A—blue, B—green, C—red), and one benign fibroma/mesothelial (pink) co-culture were monitored in real time over an 18-h period, mean 15-min impedance readings with lower standard deviation are shown; *n* = 2 wells/sample of a representative experiment are shown. (**C**) Mesothelial clearance. Parallel assays demonstrating mesothelial clearance by the three patient-derived ovarian cancer spheroids (A–C) but not benign fibroma spheroids over 48 h with a representative image are shown. (**D**) RTCA adhesion and proliferation. The adhesion and proliferation of patient-derived ovarian cancer cells (A–C) and the benign control on uncoated and fibronectin-coated wells was measured by RTCA assay. Samples were monitored over a 13-h period with mean 5-min impedance and lower standard deviation shown; *n* = 2 wells/sample of one representative experiment.

We first established the invasive properties of ascites-derived OC cells, using a novel invasion assay that allows the real-time monitoring of spheroid attachment and invasion through a mesothelial monolayer in vitro (Figure 1A) [7]. In this model, OC spheroids are overlaid onto a mesothelial monolayer, and subsequently established on a matrigel matrix in a CIM-16 Real-Time Cell Analysis (RTCA) plate. Spheroid attachment and invasion through the mesothelial monolayer/matrix are monitored in real time using an xCELLigence instrument, providing a dynamic real-time snapshot of invasive cell behaviour. We have validated this model extensively, specifically for the analysis of the earliest time-points occurring during OC cell invasion in vitro [7,8].

Spheroids from patients with either benign (ovarian fibroma) or malignant high-grade serous ovarian cancer (HGSC) disease (three individual patient derived HGSC samples de-identified and labelled A, B, and C) were isolated from ascites fluid and assessed for invasive capacity (Figure 1B). Within four hours, all of the malignant HGSC cells had rapidly invaded through the mesothelial monolayer. We deemed this period the early invasive window. By contrast, spheroids obtained from a patient with benign fibroma failed to disrupt the mesothelial monolayer. Thus, the onset of cancer cell invasion occurred rapidly upon contacting a mesothelial monolayer in vitro.

### 2.2. Adhesion and Proliferation Do Not Predict the Invasive Capacity of Cells

Metastatic OC cells interact with the mesothelial monolayer lining the peritoneal cavity and organs, invading and attaching to the underlying matrix to establish secondary nodules [2,3,4]. Using primary ascites-derived tumour cells, we assessed the mesothelial displacement and the emergence of invasive filopodia from spheroids in vitro over an extended timeframe. On assay commencement, spheroids from benign or malignant samples were of similar size and displayed no apparent morphological differences (Figure 1C). The extensive outgrowth of membrane protrusions and clearance of the underlying mesothelial layer occurred within 24 h for all of the malignant samples; by contrast, benign spheroids did not display any visible evidence of membrane outgrowth or invasion. We conducted RTCA proliferation and adhesion assays, with impedance readings taken every 5 min for 8 h (cell adhesion), and subsequently every 15 min for 24 h (cell proliferation) to assess whether the lack of invasion was not due to failed adhesion or reduced cell proliferation. Indeed, benign cells displayed comparatively elevated adherence to uncoated and fibronectin-coated culture plates and achieved a higher proliferative index than malignant cell samples in RTCA (Figure 1D). These data demonstrate that only malignant cells exhibited invasive capacity, and that invasive potential cannot be predicted from the adhesive or proliferative capacity of cells in vitro.

### 2.3. Proteomic Profiling Identifies Proteins Unique to the Invasion Interface

**Figure 2:**
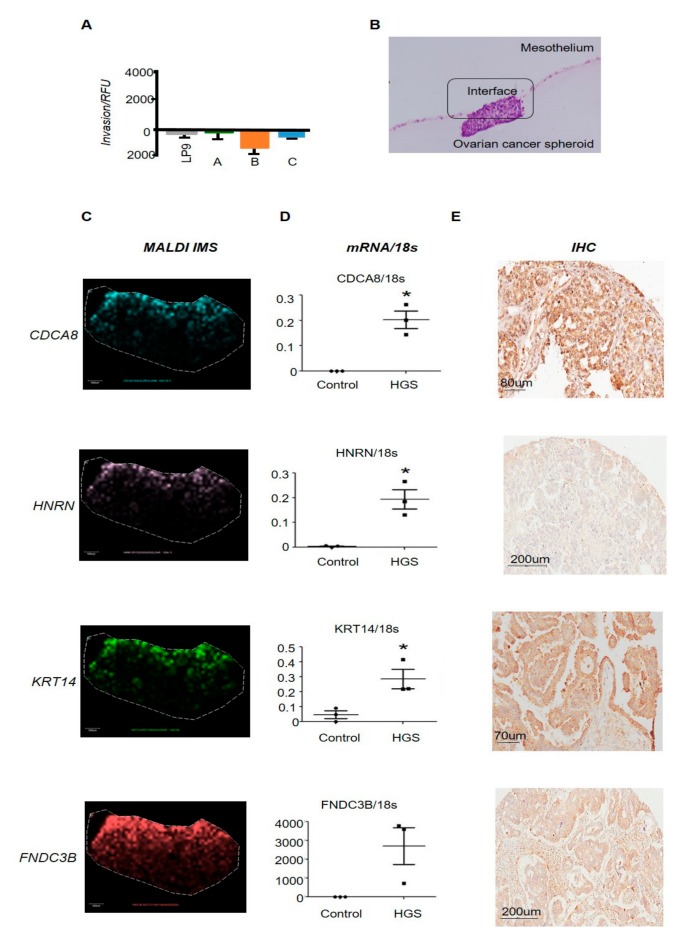
(**A**) Parallel endpoint Boyden chamber assays. Boyden chamber assays using labelled mesothelial cells overlaid with individual patient-derived ovarian cancer spheroids; we observe no invasion of the mesothelial cells at MALDI imaging collection points; *n* = 3 wells/sample of one representative experiment. (**B**) Haemotoxylin and Eosin (H&E) staining of the invasive interface. H&E staining identifying the invading interface of ovarian cancer spheroid mesothelial co-cultures and the interface used for MALDI imaging mass spectrometry (IMS). (**C**) MALDI IMS of the invading interface. MALDI IMS identifies: CDCA8, HNRN, keratin-14 (KRT14) and FNDC3B expressed at the invading interface of ovarian/mesothelial co-cultures. (**D**) Representative qRT-PCR of MALDI identified candidates using fresh-frozen confirmed primary high-grade serous ovarian tumours or normal whole ovary (*n* = 3/group) where individual data points represent individual patient samples. (**E**) Representative IHC of individual MALDI identified candidates in HGSC primary ovarian samples.

No prior studies have examined proteins directly at the interface between actively invading cancer cells and the mesothelium. To assess invasion-related protein abundance and localisation, spheroid/mesothelial co-cultures were harvested following attachment to the mesothelium, but prior to the onset of invasion (as determined by RTCA assay). Parallel endpoint Boyden chamber assays were used to confirm that mesothelial attachment but not invasion had occurred in samples used for MALDI IMS analyses (Figure 2A).

Cell–spheroid interface cultures were embedded in agarose, sectioned, and the interface was located by immunohistochemistry (IHC) (Figure 2B) and analysed by IMS. We combined the spatial localisation of intact peptides from MALDI IMS and peptide sequence information from LC-MALDI-MS/MS (Supplementary Materials Table S1). LC-MALDI identified 26 proteins, which were uniquely present in co-cultures containing malignant but not benign spheroids, at the spheroid/mesothelial interface. Amongst these were several proteins previously associated with OC (e.g., *HSP90*, *AMH,* and *OSM*) [9,10], suggesting that they may play important roles during the early stages of invasion. We further restricted our analyses to (i) include only those proteins identified in every malignant HGSC sample, and (ii) exclude proteins that were also identified in the mesothelial cell monolayer. Four proteins (KRT14, Hornerin [HRNR], Cell Division Cycle Associated 8 [CDCA8], and Fibronectin Type III Domain Containing 3B [FNDC3B]) were identified as unique to all patient HGSC cells at the cancer–mesothelial interface following this high-stringency approach (Figure 2C–E). Immunostaining on tissue micro-arrays (TMAs) and RT-PCR on fresh frozen primary tissue was used to confirm candidate expression and localisation in independent tumour tissue compared to histologically normal ovarian tissue (Figure 2D,E).

### 2.4. KRT14 at the Invasive Interface Is Required for Ovarian Cancer Cell Invasion

**Figure 3:**
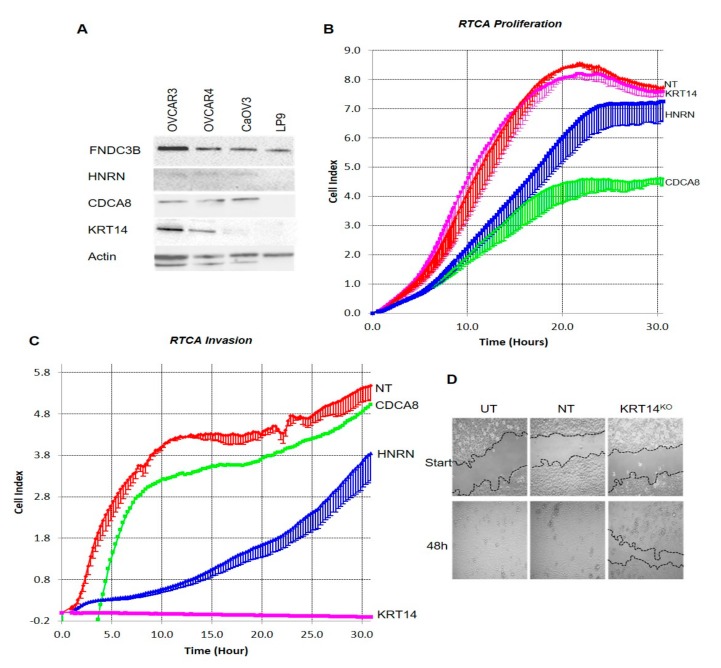
(**A**) Western blot analyses demonstrating the endogenous expression of MALDI identified candidates FNDC3B, HNRN, CDCA8, or KRT14 in ovarian cancer cell lines or the mesothelium LP-9. (**B**) RTCA proliferation assays. Representative results from OVCAR4 real-time proliferation assays conducted in complete culture medium. Data are mean impedance readings from triplicate wells taken every 15 min over a 30 h assay period with lower standard deviation shown, where the non-targeting control is NT and individual CRISPR gene knock-outs are indicated. (**C**) RTCA invasion assays. Representative results from OVCAR4 real-time invasion assays. Data are mean impedance readings from duplicate wells taken every 15 min over a 26 h assay period with lower standard deviation shown. Individual CRISPR gene knock-outs are indicated on the graph. (**D**) Wound-healing assays. Wounded OVCAR4 cell lines (UT = untreated, NT= non-targeting and KRT14^KO^ = KRT14 CRISPR knockout) were cultured in complete medium. Cells were imaged at regular intervals ranging from 0 to 48 h with the original wound area indicated by dotted lines to aid in the assessment of wound closure. Images presented are representative of the observations of three separate experiments at assay commencement and 48 h, the assay was completed on a minimum of three separate occasions.

We next examined the abundance of *HRNR*, *KRT14*, *CDCA8,* and *FNDC3B* in multiple HGSC cell lines (OVCAR3, OVCAR4, and CaOV3) [11] by Western blot. In agreement with RT-PCR data (above), HRNR, KRT14, and CDCA8 were detected in cancer cell lysates but not in the mesothelial cell controls (Figure 3A). FNDC3B was additionally detected in LP9 mesothelial cells, and was excluded from further analyses (Supplementary Materials Table S2). *KRT14*, *CDCA8,* and *HRNR* were then knocked out (CaOV3 and OVCAR4 cell lines) using CRISPR, with their specific loss confirmed in clonal populations by sequencing PCR and Western blot (Supplementary Materials Table S3 and Figure S1). The effects of functional *KRT14*, *CDCA8,* or *HRNR* loss on cell proliferation and invasion were tested by RTCA. Compared to either untreated or non-targeting controls, cells lacking *HRNR* or *CDCA8* showed significantly reduced proliferation (Figure 3B); by contrast, the loss of *KRT14* did not affect proliferation, suggesting that it was not required for tumour cell viability or growth. Both *CDCA8* and *HRNR* knock-out cells also retained invasion competency; *CDCA8* knock-out cells displayed similar invasion kinetics to untreated or non-targeting cells, whilst *HRNR* knock-out cells exhibited a lag in the onset of invasion (Figure 3C). However, cells lacking functional *KRT14* displayed a complete loss of invasive capacity with no invasion observed after 30 h (or over extended periods up to seven days—data not shown). The *KRT14*-mediated loss of invasion competence was accompanied by a parallel loss of migratory capacity in wound-healing assays, where *KRT14* knock-out (KRT14^KO^) cells failed to repair a wounded monolayer after 48 h (Figure 3D). Thus, further studies focused on *KRT14* as a key gene controlling the invasive capacity of malignant OC cells.

### 2.5. Migratory Cells Display Increased KRT14 Expression

**Figure 4:**
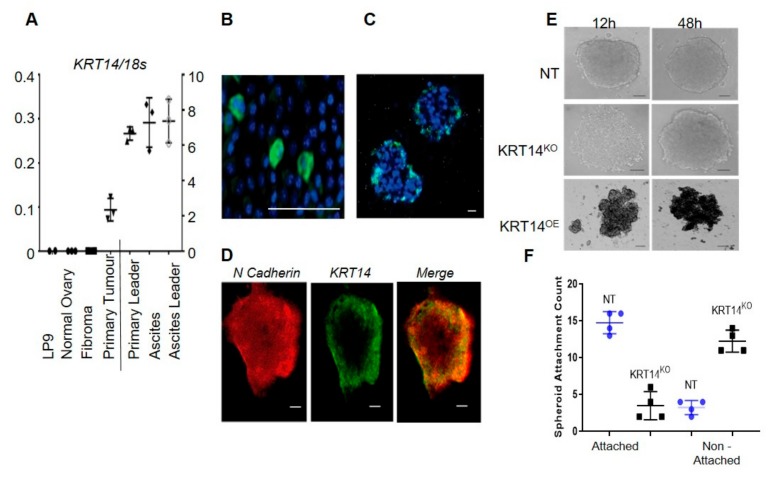
(**A**) Representative qRT-PCR of KRT14 expression in LP9 cells, whole normal ovary, benign fibroma, primary ovarian tumour, or ascites-derived cells; and migratory leader cell populations (*n* = 3 separate patient isolations where individual data points represent individual patient samples). (**B**) KRT14 expression in cultured cells by immunofluorescence. KRT14 expression was detected as a granular cytoplasmic stain expressed by a small proportion of ovarian cancer cells in the OVCAR4 line cultured as a two-dimensional (2D) monolayer. Scale bar = 100 µm. (**C**) KRT14 expression was detected at the periphery of OVCAR4 ovarian cancer cells cultured as three-dimensional (3D) spheroids with no signal detected in the core of the spheroid. Scale bar = 100 µm. (**D**) Localisation of KRT14 at the periphery of ovarian cancer spheroids by comparison to the intracellular staining observed for N-cadherin. Scale bar = 100 µm. (**E**) Vector control (NT), KRT14^KO^, and KRT14^OE^ cells were suspended in SFM/0.25% methylcellulose in U-bottom 96-well plates and imaged at regular intervals (0–48 h) to observe spheroid aggregation. Images are representative of multiple wells observed at the 13-hour and 48-hour time points. Scale bar = 100 µm. (**F**) Spheroid attachment to the target mesothelium. Vector control (NT) and KRT14^KO^ spheroids were co-cultured with the target mesothelium, and attachment was measured 6 h post-addition. The results presented are from one representative assay where data points are the attachment counts of three individual wells per cell line.

*KRT14* mRNA expression was measured in bulk cells from clinical specimens including (i) primary tumour tissue; (ii) ascites-derived HGSC cells; (iii) benign cells; (iv) histologically normal ovary; and (v) the target peritoneal cell layer LP9 alone (*n* = 3 per group). All the malignant cells expressed KRT14 at assay commencement, with no expression detected in benign fibroma, normal ovary, or LP9 mesothelial cells (Figure 4A). Cells were further seeded onto RTCA CIM plates; we detected migratory cells in the lower chamber for all the malignant samples (i.e., tumour tissue or ascites-derived), and failed to detect migratory cells isolated from benign ascites, normal controls, or LP9 cells alone. We further isolated “leader” cells from the underside of the migration plate and examined KRT14 expression. We show that KRT14 mRNA was significantly enriched in the migratory samples as compared to pre-migratory samples where the ascites-derived OC cell population displayed the highest levels of KRT14 mRNA. Together, the data suggest that whilst KRT14 has no effect on cell viability or proliferation, it is specifically required to maintain the invasive potential of the migratory cancer cell subset in vitro, and its expression is significantly enriched in actively migrating cells.

### 2.6. KRT14 Is Restricted to a Sub-Population of HGSC Cells that Influence Spheroid Assembly and Adhesion to the Mesothelium

We next determined the KRT14 abundance and localisation in OC cells (both immortalised and primary ascites-derived) by immunostaining. In a monolayer culture, KRT14 was restricted to a few isolated cells (Figure 4B), whilst spheroids cultured under low adhesion conditions showed KRT14 immunostaining exclusively localised to the outer spheroid rim (Figure 4C). The absence of internal KRT14 staining was not due to the occlusion of antibodies from the spheroid, since an anti-N-cadherin antibody penetrated effectively to stain the entire spheroid (Figure 4D). We also assessed whether KRT14 expression was required for spheroid formation in vitro by examining KRT14^KO^ and KRT14 overexpression (KRT14^OE^) lines. Wild-type OVCAR4 cells, and cells transfected with non-targeting CRISPR control, formed spheroids after 12 h in low adhesion culture; by contrast, KRT14 knock-out cells remained largely dispersed after 12 h. However, extended incubation (48 h) resulted in the formation of spheroids that were of a comparable size and morphology to the control (Figure 4E). Conversely, KRT14^OE^ lines rapidly formed dense and compact spheres and demonstrated visible outgrowth from the original sphere, which was evident following a 12 h culture period. All the spheroids exhibited uniform viability (data not shown). Vector control (NT) and KRT14^KO^ spheroids were co-cultured with the target mesothelium, and attachment was measured 6 h post-addition. Compared to untreated controls, KRT14^KO^ spheroids had significantly reduced ability to adhere to a mesothelial monolayer in vitro (Figure 4F).

### 2.7. KRT14^+^ Cells Lead Invadopodia Formation and Mesothelial Clearance

**Figure 5:**
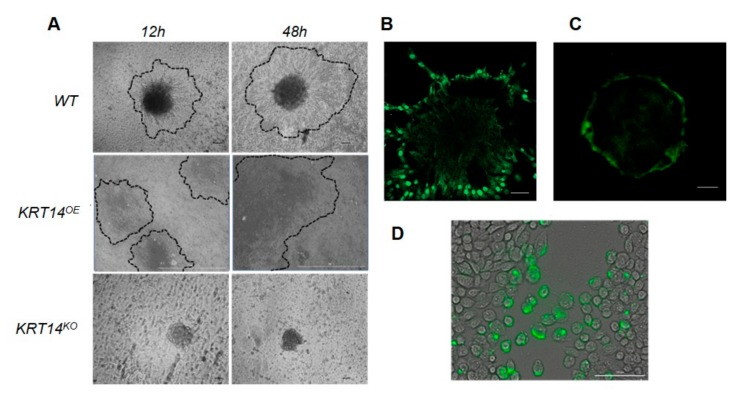
(**A**) Mesothelial displacement assays. Ovarian cancer spheroids were generated from wild-type (WT), KRT14^KO^ and KRT14^OE^ lines and overlaid onto a confluent layer of mesothelial cells and imaged by light microscope. Representative images following overnight and 48-h co-culture are presented where dotted lines indicate spheroid outgrowth and mesothelial displacement. (**B**,**C**) Embedding and outgrowth assays. OVCAR4 ovarian cancer spheroids were formed and overlaid into (**B**) Collagen I (24 h) or (**C**) Matrigel (48 h) samples, and subsequently fixed and stained for KRT14 expression. (**D**) Wound-healing assays OVCAR4 cells were grown as a monolayer, cells were wounded, fixed, and stained for KRT14 expression at time-points preceding total wound closure. Images from a representative well are shown with scale bars = 100 µm.

When inoculated onto a mesothelial monolayer, wild-type HGSC spheroids exhibited outgrowth, mesothelial clearance, and extensive deposition and migration within 24 h (Figure 5A). Cells overexpressing KRT14 rapidly dispersed and displaced the mesothelial layer; by contrast, KRT14^KO^ cells failed to disrupt the mesothelial monolayer. To examine whether the matrix type affected invasion, spheroids (both cell lines and ascites-derived HGSC cells) were embedded into either Matrigel or collagen I matrices and monitored for invadopodia outgrowth over time. KRT14^+^ invadopodia emerged from wild-type spheroids after 12 h in a collagen I matrix, but required 48 to 72 h (dependent on cell type) before becoming evident in Matrigel (Figure 5B,C). Immunostaining showed that KRT14^+^ cells were specifically localised to the invadopodia, with non-invading spheroid core cells maintaining a KRT14^−^ phenotype. This was confirmed in monolayer scratch assays, where KRT14^+^ cells were localised specifically at the areas of wound closure (Figure 5D). Consistent with their lack of invasive and migratory capacity, KRT14^KO^ cells failed to form visible invadopodia in either matrix (data not shown). Together, the data suggest that KRT14 expression is a feature of actively invading and migrating cells, and that its loss significantly impedes the ability of spheroids to displace the mesothelium and disseminate during tumour outgrowth.

### 2.8. KRT14 Is Associated with Tumour Stage, and Negatively Predicts Progression-Free Survival for Ovarian Cancer Patients

**Figure 6:**
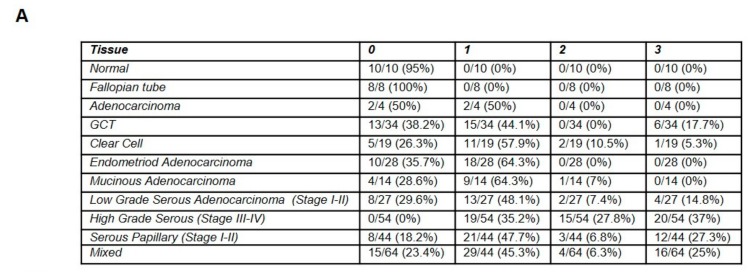

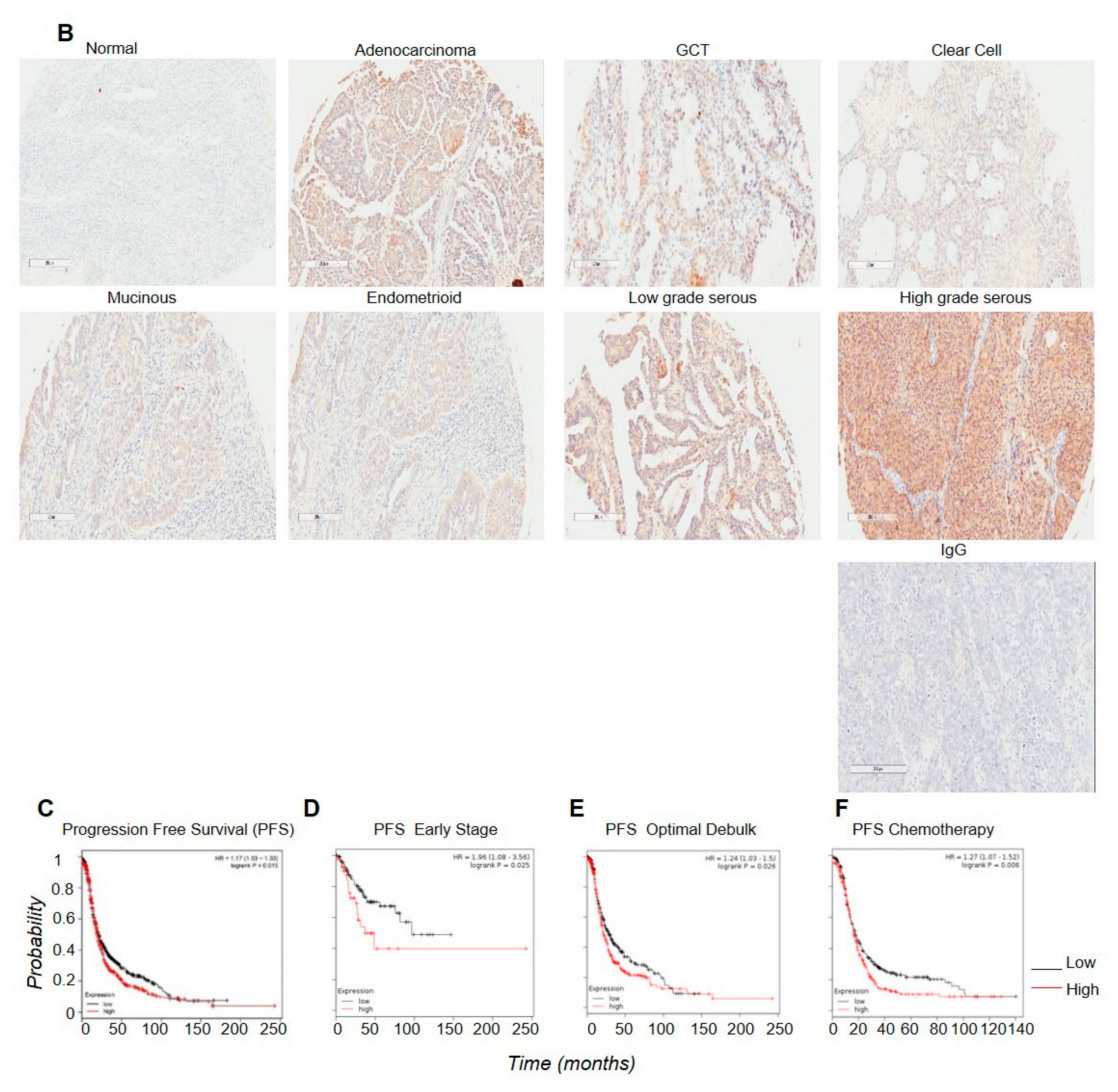
(**A**) Table of the immunohistochemical assessment of KRT14 expression in ovarian cancer tissue microarrays. Staining intensity was scored according to 0 (no stain), 1 (low), 2 (medium), or 3 (high). (**B**) Representative immunohistochemical staining of KRT14 in the various ovarian cancer subtypes (as labelled in figure) from TMA assessments. Scale bar = 200 µm. (**C**–**F**) Association of KRT14 expression with progression-free survival (PFS) according to (**C**) overall PFS (HR 1.17; 95% CI 1.03–1.33 *p* < 0.015; B); (**D**) early stage (I/II) diagnosis (HR 1.96; 95% CI 1.08–3.56 *p* < 0.025); (**E**) following platinum and taxol-based chemotherapy (HR 1.27; 95% CI 1.07–1.51 *p* < 0.006); and (**F**) following optimal debulk (HR 1.24; 95% CI 1.03–1.5 *p* < 0.026).

To establish the clinical relevance of KRT14 expression, tissue microarrays (*n* = 292) comprising multiple histological subtypes, grades, and stages of OCs, as well as normal ovary and fallopian tube sections, were stained for KRT14 abundance and localisation (Supplementary Materials Table S4 and Figure S2; Antibody Specificity Supplementary Materials Figure S3). KRT14 expression was generally not detected in normal ovary (5%, 1/20) or fallopian tube (0%, 0/8) tissue, but was universally expressed by all OC subtypes examined (Figure 6A). Staining was localised to the tumour epithelium, with little evidence of KRT14 in stromal tissue (Figure 6B) (Supplementary Materials Tables S5 and S6). In particular, KRT14 was detected in 100% of HGSC tissues, and was significantly elevated compared to normal ovary (*p* = 0.0362, unpaired *t*-test with Tukey’s post hoc).

Potential associations between KRT14 expression and patient prognosis were then interrogated in 15 publically available OC data sets (http://www.cbioportal.org/) [12]. High *KRT14* expression was associated with reduced progression-free survival (PFS) (HR 1.17; 95% CI 1.03–1.33 *p* < 0.015) (Figure 6C), particularly for patients diagnosed with early-stage (stages I–II) disease (HR 1.96; 95% CI 1.08–3.56 *p* < 0.025) (Figure 6D). High KRT14 expression was also associated with reduced PFS following platinum and taxol-based chemotherapy (HR 1.27; 95% CI 1.07–1.51 *p* < 0.006) (Figure 6E), and was a negative predictor of PFS following optimal debulk (HR 1.24; 95% CI 1.03–1.5 *p* < 0.026) (Figure 6F). Accordingly, patients with a shallow deletion in *KRT14* were more sensitive to chemotherapy and exhibited improved response to primary therapy. Thus, *KRT14* expression is an independent predictor of prognosis for patients with high-grade serous OCs.

## 3. Discussion

Successful adhesion to the peritoneal mesothelium, and subsequent mesothelial clearance exposing the underlying stroma, are key rate-limiting steps in the metastatic spread of OCs. Whilst mesothelial attachment and clearance by OC cells have been clearly demonstrated [6], our understanding of the molecular events driving these processes remains poor. We have combined our novel in vitro invasion model with MALDI IMS to establish a preliminary “snapshot” of the events occurring at the invasive interface of OCs. This is the first demonstration of MALDI IMS to image the early invasion interface, allowing the identification of potential mediators that control the early steps of cancer cell attachment and invasion to a mesothelial layer. Amongst the proteins identified were several targets already in clinical trials for OC treatment, validating the workflow and demonstrating its potential to identify biologically relevant events in vitro.

Of particular interest, we identified KRT14 as an absolute determinant of invasive potential in OC cells. KRT14 is an intermediate filament protein that is typically expressed by basal epithelial progenitor cells residing in the epithelial niches of healthy adult tissues [13]. These cells exhibit migratory behaviour and self-renewal, give rise to multiple differentiated progeny, and regulate branching architecture during organogenesis [14,15,16,17]. Whilst KRT14 was required for invasion, its absence had no impact on cell viability or proliferation; thus, it is likely that KRT14 plays a specific role during invasion, beyond the basic maintenance of cytoskeletal integrity. KRT14-expressing cancer cells were observed in several invasive tumour types [13,14,18,19,20,21], and are specifically associated with invasive cancer cells but absent from the non-invasive component [22,23,24]. Recent breast cancer studies clearly demonstrated that the KRT14-expressing sub-population of cells regulated the formation of polyclonal tumour deposits both in vitro and in vivo [20,21]. Our data extend this paradigm to OCs, suggesting that the KRT14-expressing sub-population of OC cells likely control mesothelial attachment and collective invasion into the underlying stroma.

The passive dissemination of cancer spheroids within the peritoneal cavity is the primary mode of OC spread, and facilitates the “collective invasion” of polyclonal tumour cell aggregates into the underlying matrix [25]. Collective invasion is common to many solid tumours [20,21,26,27], and is typically controlled by a functionally distinct subset of “leader cells” (LCs) [1]. On ECM contact: LCs form integrin adhesions required for attachment [6]; differentiate to a highly motile phenotype expressing selected basal epithelial genes [20,21,26,28,29]; and coordinate the complex cell–cell and cell–matrix interactions required to invade and establish new tumour deposits [20,21,26]. Despite an absolute requirement for integrin-mediated attachment during invasion [6], the LC population in EOCs has not been established. KRT14 filament nucleation occurs directly adjacent to newly formed focal adhesions [30], suggesting that it plays an important role in adhesion formation or stability. Similar to breast and bladder cancers [14,20,21], our data indicate that KRT14 expression may also mark the LC population in OCs.

We also observed invasion by KRT14-expressing cancer cells on a collagen I matrix, which was in agreement with previous data [31]. Mesothelial cells secrete multiple factors to promote the attachment and migration of cancer cells, and ECM proteins (e.g., fibronectin, laminin, collagen) are key regulators of these interactions [3,6,31]. Altered collagen fiber assembly and density has been demonstrated in both breast and ovarian malignancies [32,33], whilst the preferential invasion of OVCA433 cells on fibronectin [6] has also been observed; multiple integrin (e.g., α2, α5, α6, β1) and adhesion proteins (e.g., CD44) have been implicated in the process [34]. It is interesting to note that whilst circulating tumour cells are present in OC patients [35], these cancers remain largely confined to the peritoneum [4]. It is likely that the combination of ECM composition and effector molecules produced by mesothelial cells are an active participant in KRT14^+^ cell-mediated invasion, and establish a microenvironment that is permissive for tumour deposition and invasion within the peritoneal cavity. A limitation to the current study is the use of benign fibromas derived from the ascites as non-malignant/non-invasive controls. These samples comprise the mesenchymal (fibroblast) component with limited epithelial cells. As additional controls cells derived from the whole “normal” ovary, the fallopian tube or short-term cultures of each could be examined. These lines as controls add further complexity to the analyses, as the whole normal ovary contains stromal complements, and the ovarian surface epithelium (OSE) represents only a fraction of the total ovary.

Whilst the loss of KRT14 impaired spheroid assembly kinetics, it did not prevent their formation; in agreement, non-invasive benign-derived cells that did not express KRT14 also formed spheres, demonstrating that KRT14 is not required for spheroid formation. Interestingly, benign cells also displayed greater adhesive potential than malignant cells, despite lacking KRT14 expression. Thus, adhesive capacity alone is not an indicator of invasive behaviour. We also observed the localisation of KRT14 to the outer rim of free-floating malignant spheroids, suggesting they may be maintained in a primed, invasion-ready state. Thus, it is likely that malignant OC spheroids are able to immediately initiate attachment and invasion on contacting a permissive environment.

KRT14 expression was negatively associated with progression-free survival and response to therapy in OC patients. Similar correlations have been observed in other tumour types [13,14,18,19,20,21,36], where the KRT14-expressing cell subset is increased in urothelial cancers in response to chemotherapy. However, the restricted occurrence of KRT14 expression to a sub-population of cells likely masks their overall influence in publically available datasets; thus, the influence of KRT14 expression may be more profound than indicated. It is tempting to conjecture that the enrichment of KRT14-expressing cells may occur over time, following repeated rounds of chemotherapy; indeed, the chemo-enrichment of KRT14^+^ cells has been reported in bladder cancers [37]. This, in turn, would enhance tumourigenic potential, and suggest a reason for the overwhelming rates of relapse in patients with HGSC.

## 4. Materials and Methods

### 4.1. Cell Culture

Cell lines NIH-OVCAR-4 and CaOV-3 #HTB-75 were purchased from American Type Culture Collection (ATCC). OVCAR-4 cells were maintained in Roswell Park Memorial Institute medium-1640 (RPMI) (Life Technologies, Mulgrave, Australia 21870092); CaOV-3 cells were maintained in Dulbecco’s modified eagle medium (DMEM) (Thermo Scientific, Scoresby, Australia #11965118) each supplemented with 10% fetal calf serum (FCS) (Thermo Scientific, Scoresby, Australia #16000044) and 1% penicillin-streptomycin (Thermo Scientific, #15240062). The human mesothelial cell line LP9 (Coriell Institute Cell Repository, Camden, United States of America#AG07086) was maintained in HamsF12/199 medium with 10% FCS, 1% penicillin-streptomycin, 10 ng/mL of epidermal growth factor (EGF), and 0.4 ug/mL of hydrocortisone. All the lines were maintained at 37 °C with 5% CO_2_, and cell viability counts were conducted prior to the commencement of all the assays using the Countess^®^ II. Non-adherent tumour cells were obtained from the malignant ascites following patient consent using our established purification methods [38] and confirmed as HGS by pathology assessment; patient numbers were removed and lines were relabelled as A, B or C, maintained under low adhesive conditions in MCDB: F12 mediµ and 10% FCS prior to analysis.

CRISPR KRT14 targeted disruption and KRT14 overexpression. CRISPR-mediated gene silencing was performed as per the Zhang lab protocol [39], using three guide strands per gene (Supplementary Materials Table S5). Cells were transfected with guide strands (1–3), a non-targeting control, or the KRT14 overexpression construct KRT14^OE^ (Origene, Rockville, United States of America#RC214907) using Lipofectamine^®^ 2000 Transfection Reagent (Invitrogen, Carlsbad, United States of America #11668019) in DMEM as per the manufacturer’s protocol. Following transfection and a 12-h recovery period, cells were sub-cultured into selective medium and maintained under selective pressure by the addition of 1 µg/mL of puromycin (Sigma-Aldrich, Castle Hill, Australia #P8833) or Geneticin™ Selective Antibiotic (G418 Sulfate) (Life Technologies, Mulgrave, Australia #10131-035). Cells were subject to limited dilution where the selection medium was replaced every two days for approximately two weeks. Individual colonies were expanded, and knockdown of the target gene was measured by Western blot analyses and verified by Sanger sequencing.

### 4.2. Human Tissue Arrays and Immunohistochemistry

Immunohistochemistry was performed on TMA sections purchased from USBIOMAX (#ov2085, #ov20811) or generated in house (fallopian tube) as previously described [8, Rainczuk, 2013 #407] (Supplementary Materials Figure S2). For antigen retrieval, sections were incubated for 10 min in 50 mM of glycine (pH 3.5) at 90 °C. Sections were incubated overnight at 4 °C with Rb-KRT14 antibody (1:500, Sigma, SAB4501657), Gt-Hornerin (1:500, ab78909), Rb-Borealin/CDCA8 (1:150 sc-130705), in 0.1% Bovine Serum Albumin (BSA)/PBS. Subsequent steps were performed at room temperature, with PBS washes between incubations. Sections were incubated with goat anti-rabbit Immunoglobulin G(IgG) peroxidase conjugate (1:1000, Dako, Glostrup, Denmark, PO448) or biotinylated rabbit anti-goat IgG antibody (1:1000, Vector Laboratories, BA-5000) for 1 h, followed by a Vectastain Elite ABC kit according to the manufacturer’s instructions (Vector Laboratories, Burlingame, CA, USA). Antibody binding was detected as a brown precipitate after development with 3,3′-diaminobenzidine tetrahydrochloride; Harris hematoxylin was used as counterstain. The sections were mounted under glass coverslips in Depex (BDH Laboratory Supplies, Poole, United Kingdom). Positive immunostaining was assessed relative to parallel sections exposed to an isotype (IgG) control. Immunostaining in tumour and stromal tissue was assessed using Aperio ImageScope (v 12.3.3) as described [40].

### 4.3. Western Blot Analyses

SDS-PAGE and Western blotting were performed as previously described [40]. Blots were probed using antibodies against KRT14 (1:1000, SAB4501657), FNDC3B (1:1000 sc-393997), Hornerin (1:500 ab78909), Borealin/CDCA8 (1:800 sc-130705), and β-actin (1:20,000; Sigma-Aldrich, Castle Hill, Australia). Secondary antibodies were horseradish peroxidase (HRP)-conjugated goat anti-mouse, anti-rabbit, and donkey anti-goat (1:50,000; Merck Millipore, Kilsyth, Australia) were used [41]. Protein bands were detected using Clarity Western enhanced Chemiluminescent (ECL) blotting substrate (Biorad #1705061) and visualised by ChemiDoc™ MP (Bio-Rad, #1708280).

### 4.4. xCELLigence Real-Time Cell Analyses (RTCA)

Real-time cell analyses (RTCA) were conducted using an xCELLigence RTCA SP 96-well instrument (ACEA Biosciences). Cell lines were synchronised in G_o_ by overnight incubation in serum-free media prior to commencement. For proliferation assays, cells were seeded at 0.5 × 10^3^ cells/0.14 mL/ per well, and impedance readings were taken every 5 min for 8 h (cell adhesion), and subsequently every 15 min for 24 h (cell proliferation). For invasion assays, the upper chamber of a CIM-16 well plate was coated with Matrigel matrix (1:10 in SFM; BD Biosciences, North Ryde, Australia). Cells were seeded into the upper chamber (as above), with media ± 10% FBS added to the lower chamber. All the assays were performed in duplicate or triplicate, in at least three independent experiments.

### 4.5. Peritoneal Microenvironment Model

To establish a model of the peritoneal microenvironment, two-chamber RTCA CIM plate wells were prepared by coating the upper chamber with Matrigel (1:10 in serum free media [SFM]; BD Biosciences); then, 7 × 10^4^ LP9 cells cells/well were added and monitored until a confluent monolayer was formed. Spheroids (from fresh patient ascites; 10 spheres/well) were seeded in SFM in the upper chamber, and media ± 10% fetal bovine serum (FBS) added to the lower chamber. Real-time readings were used to determine the optimal time(s) to use as collection points for MALDI imaging analysis, with all the samples prepared in duplicate or triplicate wells per experiment. As an additional control, concurrent endpoint invasion assays were conducted in parallel using modified Boyden chambers. In this case, mesothelial cells were labelled using Cell Trace™ Carboxyfluorescein succinimidyl ester (CFSE) prior to inoculation with OC spheroids. Mesothelial invasion was assessed according to the retraction of CSFE-labelled mesothelial cells underneath spheroids, using a Cytation™ 3 Multimode Imager (BioTek Instruments, Winooski, VT, USA).

### 4.6. Preparation of Samples for MALDI-IMS

Spheroid–mesothelial interfaces were co-cultured on Thermanox™ sectionable coverslips, and harvested following attachment to the mesothelium, but prior to the onset of invasion (as determined by RTCA assay). Samples were sectioned at 5 μm, and the invasive interface was located by H&E staining on periodic sections. Once identified, two unstained sections through the interface were placed on an indium–tin–oxide (ITO) slide for MALDI processing (Bruker Pty Ltd, Preston, Australia).

### 4.7. MALDI IMS

Tryptic peptides at the OC–spheroid–mesothelial interface were identified using the ImageID workflow (Bruker). Trypsin was applied to serial tissue sections by nebulisation using an ImagePrep spray device (Bruker). Samples were digested in a humidified chamber for 90 min, after which peptides were extracted and purified. LC-MALDI analysis was performed using an ultrafleXtreme MALDI-TOF/TOF (Bruker) and a Dionex Ultimate 3000 RSLC system (Thermo Scientific, Scoresby, Australia) as described [42]. MALDI acquisition of a subsequent digested serial sections was performed using flexImaging 4.1 (Bruker) as previously described [42]. LC-MALDI data and MALDI imaging data were compared and filtered using ImageID software, mass peaks were matched between imaging data, and LC-MALDI analysis with mass tolerance was calculated by ImageID software.

### 4.8. Methylcellulose Overlay and Sphere Formation

OC cells were dissociated by trypsinisation and resuspended in complete cell culture medium (minimum viability of 98%). A total of 2500 cells per sphere were overlaid in 0.25% methylcellulose (Sigma Aldrich, Castle Hill, Australia) in serum-free medium, and seeded into a single well of a 96-well CELLSTAR^®^ U-bottom Suspension Culture Plate (Greiner Bio One, Interpath Services PTY, Vic, Australia). Spheroid aggregation and formation for each line was observed and imaged at regular intervals using a light microscope. Formed spheres were harvested using a wide bore tip and centrifugation.

### 4.9. Mesothelial Displacement Assay

The human mesothelial cell line LP9 was seeded as above and incubated at 37 °C until a confluent monolayer was formed. A total of 16 spheres per well were seeded onto the confluent mesothelial monolayer. Mesothelial displacement and outgrowth were imaged at regular intervals using phase-contrast microscopy.

### 4.10. In Vitro Wound Repair Assay

OC cells (KRT14^KO^, WT and NT control) were grown in complete medium to confluency in a 12-well plate and serum-starved to synchronise at G_0_. The following day the cell culture medium was removed, and cell monolayers were wounded by scraping with a pipette tip attached to suction. Non-adherent cells were removed by gentle washes with PBS, and the complete growth medium was replaced. The wound area was imaged under a phase microscope (Leica) at regular intervals ranging from 0 to 72 h. Wound closure was measured in an image series using AnalySIS LS Research Software (Olympus, Notting Hill, Australia). Experiments were repeated in triplicate with at least six wound areas observed per growth condition.

### 4.11. Matrigel and Collagen I Outgrowth Assay: Staining and Imaging

Our protocol was adapted from Nguyen-Ngoc [43]. Briefly, OC spheroids were collected to yield a suspension of six spheres per matrix. Spheres were embedded in either 3D Matrigel (354230; BD Biosciences) or rat-tail collagen I (354236; BD Biosciences). Cultures were set up in eight-well plates on coverglass slides (94.6190.802, Starstedt) as per [43]. For antibody staining, spheres cultured in either Matrigel or collagen I were fixed with 4% (wt/vol) paraformaldehyde for 30 min, rinsed twice in PBS for 10 min, permeabilised with 0.5% (vol/vol) Triton X-100 in PBS for 20 min, and rinsed twice in PBS for 10 min and blocked in 10% (vol/vol) FBS in PBS for 2 h at room temperature then incubated with primary antibody (1:1000, Anti-N Cadherin antibody [5D5] ab98952 AbCam and 1:500, KRT14) overnight at 4 °C. The following day, samples were washed three times with PBS and incubated with secondary antibodies: 1:2000, goat anti-rabbit IgG Alexa 467, ab150083, and 1:2000 goat anti-mouse IgG Alexa 488, ab150117) for 3 h at room temperature, and then rinsed three times in PBS for 10 min. Samples were imaged using a Cytation™ 3 Mulitmode Imager (BioTek Instruments, Winooski USA) with Gen5 Image + software or Nikon C1 Confocal microscope (Monash Micro Imaging Facility, Monash).

### 4.12. Real-Time PCR

Total RNA was extracted from primary high-grade serous ovarian tumours (*n* = 3) and the whole normal ovary (*n* = 3) using the Tissue Lyser LT system with 5-mm stainless-steel beads and the RNeasy Mini Kit (Qiagen). The total RNA was extracted from OC cell lines OVCAR4 and CaOV3, and then grown as monolayers or spheroids (KRT14^KO^ and wild-type lines), ascites-derived OC (*n* = 3) or benign fibroma (*n* = 2) spheroids, and the target peritoneal cell layer LP-9 using the RNeasy Mini Kit as per the manufacturer’s protocol. Sense and antisense oligonucleotide primers to KRT14, HNRN, FNDC3B, 18S, and CDCA8 were designed against published human sequences and verified as previously described [44]. cDNA was synthesised using Superscript III reverse transcriptase (Life Technologies, Grand Island, NY). Real-time PCR samples were prepared to a final volume of 10 µL using the Applied Biosystems ABI SYBR mix (Scoresby, Victoria, Australia). Quantitative real-time PCR was completed as previously described [44] using the Applied Biosystems ABI 7900 HT Fast real-time machine with all the reactions performed in triplicate. Yields were converted to femtograms based on the standard curve for each PCR product, and the resultant mRNA levels were normalised to the 18S mRNA level per sample.

### 4.13. Statistical Analyses

Statistical analyses were conducted using GraphPad Prism (Version 8; GraphPad Software Inc., San Diego, CA). For data derived from cell assays, means were compared using one-way or two-way ANOVA with Bonferroni’s, Dunnett’s, or Tukey’s post hoc tests, as indicated. To determine whether mRNA expression significantly differed between samples, a Mann–Whitney U-test or unpaired *t*-test was performed. Means were considered significantly different if *p* < 0.05. All the experiments were independently repeated at least three times.

### 4.14. Kaplan–Meier Curves

The Kaplan–Meier online plotter tool (http://kmplot.com/analysis/) was used to generate survival curves using mRNA data from serous OC patients from 15 public OC data sets where the best cut-off values were auto-selected by the plotter tool, and the log rank, *p* value, and hazard ratio (and 95% confidence intervals) were calculated [12].

## 5. Conclusions

In summary, we have identified KRT14-expressing cells as a key determinant for mesothelial attachment, clearance, and subsequent invasion by OC cells. This subset of cancer cells likely represent the leader cell phenotype responsible for promoting/leading collective tumour invasion in vivo, and is associated with response to therapy and survival. Taken together, our data suggest that KRT14^+^ cells underlie invasion by OC spheroids, and are an important clinical feature of HGSC.

## Supplementary Materials

The following are available online at https://www.mdpi.com/2072-6694/11/9/1228/s1. Figure S1: Western Blot screening following CRISPR transfection and selection, Figure S2: KRT14 and H&E staining of USBiomax TMAs, Figure S3: KRT14 antibody specificity on wild type, overexpressing and K14 knockout cells—OVCAR4, Figure S4: xCELLigence Proliferation and invasion—CaOV3, Table S1: MALDI target identification, Table S2: Densitometry assessments for Western blot from figure 3A, Table S3: Western Blot Densitometry, Table S4: Patient sample details for TMA analyses, Table S5: CRISPR sgRNA sequences to target candidates.
