# Supplementary material for: Keratin-14 (KRT14) Positive Leader Cells Mediate Mesothelial Clearance and Invasion by Ovarian Cancer Cells
Source: Cancers (Basel). 2019 Aug 22;11(9):1228. doi: 10.3390/cancers11091228 (PMC6769856; doi:10.3390/cancers11091228)
Supplement: Supplementary file 1 [file cancers-11-01228-s001.pdf]

Table S1: MALDI target identification

Supplementary Table 1.

Proteins & Peptides identified by LC-MALDI-TOF/TOF as part of the ImageID workflow.

Masses identified by LC-MALDI-TOF/TOF were then correlated with peptides identified by MALDI Imaging Mass Spectrometry.

Protein Report

**Protein 1:**	Keratin, type II cytoskeletal 1 OS=Homo sapiens GN=KRT1 PE=1 SV=6		
**Accession:**	K2C1_HUMAN	**Score:**	834.14
**Database:**	SwissProt	**MW [kDa]:**	66.00
**Seq. Coverage [%]:**	17.40 %	**pI:**	8.82
		**No. of Peptides:**	5

10	20	30	40	50	60	70	80	90	100	110	120
MSRQFSSRSG	YRSGGGFSSG	SAGIINYQRR	TTSSSTRRSG	GGGGRFSSCG	GGGGSFGAGG	GFGSRSLVNL	GGSKSISISV	ARGGGRGSGF	GGGYGGGGFG	GGGFGGGGFG	GGGIGGGGFG
130	140	150	160	170	180	190	200	210	220	230	240
GFGSGGGGFG	GGGFGGGGYG	GGYGPVCPPG	GIQEVTINQS	LLQPLNVEID	PEIQKVKSRE	REQIKSLNNQ	FASFIDKVRF	LEQQNQVLQT	KWELLQQVDT	STRTHNLEPY	FESFINNLRR
250	260	270	280	290	300	310	320	330	340	350	360
RVDQLKSDQS	RLDSELKNMQ	DMVEDYRNKY	EDEINKRTNA	ENEFVTIKKD	VDGAYMTKVD	LQAKLDNLQQ	EIDFLTALYQ	AELSQMQTQI	SETNVILSMD	NNRSLDLDSI	IAEVKAQYED
370	380	390	400	410	420	430	440	450	460	470	480
IAQKSKAEAE	SLYQSKYEEL	QITAGRHGDS	VRNSKIEISE	LNRVIQRLRS	EIDNVKKQIS	NLQQSISDAE	QRGENALKDA	KNKLNDLEDA	LQQAKEDLAR	LLRDYQELMN	TKLALDLEIA
490	500	510	520	530	540	550	560	570	580	590	600
TYRTLLEGEE	SRMSGECAPN	VSVSVSTSHT	TISGGGSRGG	GGGGYGSGGS	SYGSGGGSYG	SGGGGGGGRG	SYGSGGSSYG	SGGGSYGSGG	GGGGHGSYGS	GSSSGGYRGG	SGGGGGGSSG
610	620	630	640	650							
GRGSGGGSSG	GSIGGRGSSS	GGVKSSGGSS	SVKFVSTTYS	GVTR							

Cmpd.	No. of Cmpds.	m/z meas.	Δ m/z [ppm]	z	Rt [min]	Score	Site [%]	P	Range	Sequence	Modification	Type
111	1	1475.7923	4.78	1	17.95	68.12		0	200-211	R.FLEQQNQVLQTK.W		CID
31	1	1308.6608	4.99	1	14.65	62.72		2	268-277	R.NKYEDEINKR.T		CID
121	1	1265.6501	10.22	1	18.70	76.14		0	278-288	R.TNAENEFVTIK.K		CID
40	2	2383.9526	0.33	1	15.70	343.04		0	519-549	R.GGGGGGYGSGGSSYGSGGGSYGSGGGGGGGR.G		CID
99	1	3312.3216	4.07	1	17.35	260.22		0	550-588	R.GSYGSGGSSYGSGGGSYGSGGGGGGHGSYGSGS[illegible] GYR.G		CID

Protein 2: keratin 10 (epidermolytic hyperkeratosis; keratosis palmaris et plantaris), isoform CRA_b [Homo sapiens]

**Accession:**	gi|119581085	**Score:**	325.57
**Database:**	NCBInr	**MW [kDa]:**	63.30
**Seq. Coverage [%]:**	6.30 %	**pI:**	5.00
		**No. of Peptides:**	4

10	20	30	40	50	60	70	80	90	100	110	120
MSVRYSSSKH	YSSSRSGGGG	GGGGCGGGGG	VSSLRISSSK	GSLGGGFSSG	GFSGGSFSRG	SSGGGCFGGS	SGGYGGLGGF	GGGSFRGSYG	SSSFGGSYGG	SFGGGSFGGG	SFGGGSFGGG
**130**	**140**	**150**	**160**	**170**	**180**	**190**	**200**	**210**	**220**	**230**	**240**
GFGGGGFGGG	FGGGFGGDGG	LLSGNEKVTM	QNLNDRLASY	LDKVRALEES	NYELEGKIKE	WYEKHGNSHQ	GEPRDYSKYY	KTIDDLKNQI	LNLTTDNANI	LLQIDNARLA	ADDFRLKYEN
**250**	**260**	**270**	**280**	**290**	**300**	**310**	**320**	**330**	**340**	**350**	**360**
EVALRQSVEA	DINGLRRVLD	ELTLTKADLE	MQIESLTEEL	AYLKKNHEEE	MKDLRNVSTG	DVNVEMNAAP	GVDLTQLLNN	MRSQYEQLAE	QNRKDAEAWF	NEKSKELTTE	IDNNIEQISS
**370**	**380**	**390**	**400**	**410**	**420**	**430**	**440**	**450**	**460**	**470**	**480**
YKSEITELRR	NVQALEIELQ	SQLALKQSLE	ASLAETEGRY	CVQLSQIQAQ	ISALEEQLQQ	IRAETECQNT	EYQQLLDIKI	RLENEIQTYR	SLLEGEGSSG	GGGRGGGSFG	GGYGGGSSGG
**490**	**500**	**510**	**520**	**530**	**540**	**550**	**560**	**570**	**580**	**590**	**600**
GSSGGGYGGG	HGGSSGGGYG	GGSSGGGSSG	GGYGGGSSSG	GHGGSSSGGY	GGGSSGGGGG	GYGGGSSGGG	SSSGGGYGGG	SSSGGHKSSS	SGSVGESSSK	GPRSAETSWD	TNKTRVIKTI
**610**	**620**	**630**									
IEEVAPDGRV	LSSMVESETK	KHYY									

Cmpd.	No. of Cmpds.	m/z meas.	Δ m/z [ppm]	z	Rt [min]	Score	Site [%]	P	Range	Sequence	Modification	Type
141	1	1707.8014	17.11	1	21.25	118.95		0	41-59	K.GSLGGGFSSGGFSGGSFSR.G		CID
52	1	993.4991	-0.90	1	15.70	32.81		0	238-245	K.YENEVALR.Q		CID
38	3	1365.6576	13.37	1	15.55	99.34		0	323-333	R.SQYEQLAEQNR.K		CID
35	3	1493.7592	16.66	1	15.25	74.47		1	323-334	R.SQYEQLAEQNRK.D		CID

Protein 3: Keratin, type II cytoskeletal 2 epidermal OS=Homo sapiens GN=KRT2 PE=1 SV=2

**Accession:**	K22E_HUMAN	**Score:**	230.35
**Database:**	SwissProt	**MW [kDa]:**	65.40
**Seq. Coverage [%]:**	5.80 %	**pI:**	8.85
		**No. of Peptides:**	3

Protein Report

10	20	30	40	50	60	70	80	90	100	110	120
MSCQISCKSR	GRGGGGGGFR	GFSSGSAVVS	GGSRRSTSSF	SCLSRHGGGG	GGFGGGGFGS	RSLVGLGGTK	SISISVAGGG	GGFGAAGGFG	GRGGGFGGGS	SFGGGSGFSG	GGFGGGGFGG
130	140	150	160	170	180	190	200	210	220	230	240
GRFGGFGGPG	GVGGLGGPGG	FGPGGYPGGI	HEVSVNQSLL	QPLNVKVDPE	IQNVKAQERE	QIKTLNNKFA	SFIDKVRFLE	QQNQVLQTKW	ELLQQMNVGT	RPINLEPIFQ	GYIDSLKRYL
250	260	270	280	290	300	310	320	330	340	350	360
DGLTAERTSQ	NSELNNMQDL	VEDYKKKYED	EINKRTAAEN	DFVTLKKDVD	NAYMIKVELQ	SKVDLLNQEI	EFLKVLYDAE	ISQIHQSVTD	TNVILSMDNS	RNLDLDSIIA	EVKAQYEEIA
370	380	390	400	410	420	430	440	450	460	470	480
QRSKEEAEAL	YHSKYEELQV	TVGRHGDSLK	EIKIEISELN	RVIQRLQGEI	AHVKKQCKNV	QDAIADAEQR	GEHALKDARN	KLNDLEEALQ	QAKEDLARLL	RDYQELMNVK	LALDVEIATY
490	500	510	520	530	540	550	560	570	580	590	600
RKLLEGEECR	MSGDLSSNVT	VSVTSSTISS	NVASKAAFGG	SGGRGSSSGG	GYSSGSSSYG	SGGRQSGSRG	GSGGGGSISG	GGYGSGGGSG	GRYGSGGGSK	GGSISGGGYG	SGGGKHSSGG
610	620	630	640								
GSRGGSSSGG	GYGSGGGGSS	SVKGSSGEAF	GSSVTFSFR								

Cmpd.	No. of Cmpds.	m/z meas.	Δ m/z [ppm]	z	Rt [min]	Score	Site [%]	P	Range	Sequence	Modification	Type
70	1	1320.5862	2.57	1	16.60	129.28		0	46-61	R.HGGGGGGFGGGGFGSR.S		CID
111	1	1475.7923	4.78	1	17.95	68.12		0	198-209	R.FLEQQNQVLQTK.W		CID
34	1	1107.5510	7.31	1	15.25	32.95		0	354-362	K.AQYEEIAQR.S		CID

Protein 4: hornerin [Homo sapiens]

Accession: gi|28557150

Database: NCBInr

Seq. Coverage [%]: 7.30 %

Score: 171.95

MW [kDa]: 48.60

pI: 10.10

No. of Peptides: 2

Protein Report

10	20	30	40	50	60	70	80	90	100	110	120
GSGSGHSSSY	EQHGSRSGQS	SRGEQHGSSS	GSSSSYGQHG	SGSRQSLGHG	QHGSGSGQSP	SPSRGRHGSG	SGQSSSYSPY	GSGSGWSSSR	GPYESGSGHS	SGLGHRESRS	GQSSGYGQHG
**130**	**140**	**150**	**160**	**170**	**180**	**190**	**200**	**210**	**220**	**230**	**240**
SSSGHSSTHG	QHGSTSGQSS	SCGQHGASSG	QSSSHGQHGS	GSSQSSGYGR	QGSGSGQSPG	HSQRGSGSRQ	SPSYGRHGSG	SGRSSSSGQH	GSGLGESSGF	GHHESSSGQS	SSYSQHGSGS
**250**	**260**	**270**	**280**	**290**	**300**	**310**	**320**	**330**	**340**	**350**	**360**
GHSSGYGQHG	SRSGQSSRGE	RHGSSSGSSS	HYGQHGSGSR	QSSGHGRQGS	GSGQSPSRGR	HGSGLGHSSS	HGQHGSGSGR	SSSRGPYESR	SGHSSVFGQH	ESGSGHSSAY	SQHGSGSGHF
**370**	**380**	**390**	**400**	**410**	**420**	**430**	**440**	**450**	**460**	**470**	**480**
CSQGQHGSTS	GQSSTFDQEG	SSTGQSSSYG	HRGSGSSQSS	GYGRHGAGSG	QSLSHGRHGS	GSGQSSSYGQ	HGSGSGQSSG	YSQHGSGSGQ	DGYSYCKGGS	NHDGGSSGSY	FLSFPSSTSP
**490**	**500**										
YEYVQEQRCY	FYQ										

Cmpd.	No. of Cmpds.	m/z meas.	Δ m/z [ppm]	z	Rt [min]	Score	Site [%]	P	Range	Sequence	Modification	Type
29	1	1947.8917	-5.09	1	14.05	58.67		0	45-64	R.QSLGHGQHGSGSGQSPSPSR.G		CID
30	1	1584.7156	0.41	1	14.65	113.28		0	91-106	R.GPYESGSGHSSGLGHR.E		CID

Protein 5: Chain A, Crystal Structure Of Atbag1 In Complex With Hsp70

Accession: gi|508123773 **Score:** 165.44

Database: NCBInr **MW [kDa]:** 41.50

Seq. Coverage [%]: 7.70 % **pI:** 6.39

No. of Peptides: 2

10	20	30	40	50	60	70	80	90	100	110	120
PAVGIDLGTT	YSCVGVFQHG	KVEIIANDQG	NRTTPSYVAF	TDTERLIGDA	AKNQVAMNPT	NTVFDAKRLI	GRRFDDAVVQ	SDMKHWPFMV	VNDAGRPKVQ	VEYKGETKSF	YPEEVSSMVL
**130**	**140**	**150**	**160**	**170**	**180**	**190**	**200**	**210**	**220**	**230**	**240**
TKMKEIAEAY	LGKTVTNAVV	TVPAYFNDSQ	RQATKDAGTI	AGLNVLRIIN	EPTAAAIAYG	LDKKVGAERN	VLIFDLGGGT	FDVSILTIED	GIFEVKSTAG	DTHLGGEDFD	NRMVNHFIAE
**250**	**260**	**270**	**280**	**290**	**300**	**310**	**320**	**330**	**340**	**350**	**360**
FKRKHKKDIS	ENKRAVRRLR	TACERAKRTL	SSSTQASIEI	DSLYEGIDFY	TSITRARFEE	LNADLFRGTL	DPVEKALRDA	KLDKSQIHDI	VLVGGSTRIP	KIQKLLQDFF	NGKELNKSIN
**370**	**380**										
PDEAVAYGAA	VQAAILS										

Protein Report

Cmpd.	No. of Cmpds.	m/z meas.	Δ m/z [ppm]	z	Rt [min]	Score	Site [%]	P	Range	Sequence	Modification	Type
186	1	1487.7207	13.06	1	30.85	66.25		0	33-45	R.TTPSYVAFTDTER.L		CID
175	1	1691.7357	6.01	1	28.00	99.19		0	217-232	K.STAGDTHLGGEDFDNR.M		CID

Protein 6: unnamed protein product [Homo sapiens]

Accession: gi|28193108

Database: NCBInr

Seq. Coverage [%]: 6.10 %

Score: 156.86

MW [kDa]: 49.30

pI: 5.20

No. of Peptides: 1

10	20	30	40	50	60	70	80	90	100	110	120
MGRGTKVILH	LKEDQTEYLE	ERRIKEIVKK	HSQFIGYPIT	LFVEKERDKE	VSDDEAEEKE	DKEEEKEKEE	KESEDKPEIE	DVGSDEEEEK	KDGDKKKKKK	IKEKYIDQEE	LNKTKPIWTR
130	140	150	160	170	180	190	200	210	220	230	240
NPDDITNEEY	GEFYKSLTND	WEDHLAVKHF	SVEGQLEFRA	LLFVPRRAPF	DLFENRKKKN	NIKLYVRRVF	IMDNCEELIP	EYLNFIRGVV	DSEDLPLNIS	REMLQQSKIL	KVIRKNLVKK
250	260	270	280	290	300	310	320	330	340	350	360
CLELFTELAE	DKENYKKFYE	QFSKNIKLGI	HEDSQNRKKL	SELLRYYTSA	SGDEMVSLKD	YCTRMKENQK	HIYYITGETK	DQVANSAFVE	RLRKHGLEVI	YMIEPIDEYC	VQQLKEFEGK
370	380	390	400	410	420						
TLVSVTKEGL	ELPEDEEEKK	KQEEKKTKFE	NLCKIMKDIL	EKKVEKVCEY	SIS						

Cmpd.	No. of Cmpds.	m/z meas.	Δ m/z [ppm]	z	Rt [min]	Score	Site [%]	P	Range	Sequence	Modification	Type
12	1	1833.8061	13.50	1	12.85	129.24		0	121-135	R.NPDDITNEEYGEFYK.S		CID

Protein 7: Keratin, type I cytoskeletal 14 OS=Homo sapiens GN=KRT14 PE=1 SV=4

Accession: K1C14_HUMAN

Database: SwissProt

Seq. Coverage [%]: 7.40 %

Score: 116.61

MW [kDa]: 51.50

pI: 4.94

No. of Peptides: 3

Modification(s): Carbamidomethyl

Protein Report

10	20	30	40	50	60	70	80	90	100	110	120
MTTCSRQFTS	SSSMKGSCGI	GGGIGGGSSR	ISSVLAGGSC	RAPSTYGGGL	SVSSSRFSSG	GACGLGGGYG	GGFSSSSSSF	GSGFGGGYGG	GLGAGLGGGF	GGGFAGGDGL	LVGSEKVTMQ
**130**	**140**	**150**	**160**	**170**	**180**	**190**	**200**	**210**	**220**	**230**	**240**
NLNDRLASYL	DKVRALEEAN	ADLEVKIRDW	YQRQRPAEIK	DYSPYFKTIE	DLRNKILTAT	VDNANVLLQI	DNARLAADDF	RTKYETELNL	RMSVEADING	LRRVLDELTL	ARADLEMQIE
**250**	**260**	**270**	**280**	**290**	**300**	**310**	**320**	**330**	**340**	**350**	**360**
SLKEELAYLK	KNHEEEMNAL	RGQVGGDVNV	EMDAAPGVDL	SRILNEMRDQ	YEKMAEKNRK	DAEEWFFTKT	EELNREVATN	SELVQSGKSE	ISELRRTMQN	LEIELQSQLS	MKASLENSLE
**370**	**380**	**390**	**400**	**410**	**420**	**430**	**440**	**450**	**460**	**470**	**480**
ETKGRYCMQL	AQIQEMIGSV	EEQLAQLRCE	MEQQNQEYKI	LLDVKTRLEQ	EIATYRRLLE	GEDAHLSSSQ	FSSGSQSSRD	VTSSSRQIRT	KVMDVHDGKV	VSTHEQVLRT	KN

Cmpd.	No. of Cmpds.	m/z meas.	Δ m/z [ppm]	z	Rt [min]	Score	Site [%]	P	Range	Sequence	Modification	Type
31	1	1107.4845	833.42	1	15.70	16.21		0	31-41	R.ISSVLAGGSCR.A	Carbamidomethyl: 10	CID
78	1	1425.6974	0.36	1	16.90	46.32		0	42-56	R.APSTYGGGLSVSSSR.F		CID
72	1	1122.5820	2.70	1	16.60	54.08		0	408-416	R.LEQEIATYR.R		CID

Protein 8: RecName: Full=Laminin subunit alpha-2; AltName: Full=Laminin M chain; AltName: Full=Laminin-12 subunit alpha; AltName: Full=Laminin-2 subunit alpha; AltName: Full=Laminin-4 subunit alpha; AltName: Full=Merosin heavy chain; Flags: Precursor

Accession: gi|215274259
Score: 109.48
Database: NCBInr
MW [kDa]: 343.70
Seq. Coverage [%]: 1.90 %
pI: 5.99
No. of Peptides: 0

Modification(s): Carboxymethyl, Propionamide

10	20	30	40	50	60	70	80	90	100	110	120
MPGAAGVLLL	LLLSGGLGGV	QAQRPQQQRQ	SQAHQQRGLF	PAVLNLASNA	LITTNATCGE	KGPEMYCKLV	EHVPGQPVRN	PQCRICNQNS	SNPNQRHPIT	NAIDGKNTWW	QSPSIKNGIE
130	140	150	160	170	180	190	200	210	220	230	240
YHYVTITLDL	QQVFQIAYVI	VKAANSPRPG	NWILERSLDD	VEYKPWQYHA	VTDTECLTLY	NIYPRTGPPS	YAKDDEVICT	SFYSKIHPLE	NGEIHISLIN	GRPSADDPSP	ELLEFTSARY
250	260	270	280	290	300	310	320	330	340	350	360
IRLRFQRIRT	LNADLMMFAH	KDPREIDPIV	TRRYYYSVKD	ISVGGMCICY	GHARACPLDP	ATNKSRCECE	HNTCGDSCDQ	CCPGFHQKPW	RAGTFLTKTE	CEACNCHGKA	EECYYDENVA
370	380	390	400	410	420	430	440	450	460	470	480
RRNLSLNIRG	KYIGGGVCIN	CTQNTAGINC	ETCTDGFFRP	KGVSPNYPRP	CQPCHCDPIG	SLNEVCVKDE	KHARRGLAPG	SCHCKTGFGG	VSCDRCARGY	TGYPDCKACN	CSGLGSKNED
490	500	510	520	530	540	550	560	570	580	590	600
PCFGPCICKE	NVEGGDCSRC	KSGFFNLQED	NWKGCDECFC	SGVSNRCQSS	YWTYGKIQDM	SGWYLTDLPG	RIRVAPQQDD	LDSPQQISIS	NAEARQALPH	SYYWSAPAPY	LGNKLPAVGG
610	620	630	640	650	660	670	680	690	700	710	720
QLTFTISYDL	EEEEEDTERV	LQLMIILEGN	DLSISTAQDE	VYLHPSEEHT	NVLLLKEESF	TIHGTHFPVR	RKEFMTVLAN	LKRVLLQITY	SFGMDAIFRL	SSVNLESAVS	YPTDGSIAAA
730	740	750	760	770	780	790	800	810	820	830	840
VEVCQCPPGY	TGSSCESCWP	RHRRVNGTIF	GGICEPCQCF	GHAESCDDVT	GECLNCKDHT	GGPYCDKCLP	GFYGEPTKGT	SEDCQPCACP	LNIPSNNFSP	TCHLDRSLGL	ICDGCPVGYT
850	860	870	880	890	900	910	920	930	940	950	960
GPRCERCAEG	YFGQPSVPGG	SCQPCQCNDN	LDFSIPGSCD	SLSGSCLICK	PGTTGRYCEL	CADGYFGDAV	DAKNCQPCRC	NAGGSFSEVC	HSQTGQCECR	ANVQGQRCDK	CKAGTFGLQS
970	980	990	1000	1010	1020	1030	1040	1050	1060	1070	1080
ARGCVPCNCN	SFGSKSFDCE	ESGQCWCQPG	VTGKKCDRCA	HGYFNFQEGG	CTACECSHLG	NNCDPKTGRC	ICPPNTIGEK	CSKCAPNTWG	HSITTGCKAC	NCSTVGSLDF	QCNVNTGQCN
1090	1100	1110	1120	1130	1140	1150	1160	1170	1180	1190	1200
CHPKFSGAKC	TECSRGHWNY	PRCNLCDCFL	PGTDATTCDS	ETKKCSCSDQ	TGQCTCKVNV	EGIHCDRCRP	GKFGLDAKNP	LGCSSCYCFG	TTTQCSEAKG	LIRTWVTLKA	EQTILPLVDE
1210	1220	1230	1240	1250	1260	1270	1280	1290	1300	1310	1320
ALQHTTTKGI	VFQHPEIVAH	MDLMREDLHL	EPFYWKLPEQ	FEGKKLMAYG	GKLKYAIYFE	AREETGFSTY	NPQVIIRGGT	PTHARIIVRH	MAAPLIGQLT	RHEIEMTEKE	WKYYGDDPRV
1330	1340	1350	1360	1370	1380	1390	1400	1410	1420	1430	1440
HRTVTREDFL	DILYDIHYIL	IKATYGNFMR	QSRISEISME	VAEQGRGTTM	TPPADLIEKC	DCPLGYSGLS	CEACLPGFYR	LRSQPGGRTP	GPTLGTCVPC	QCNGHSSLCD	PETSICQNCQ
1450	1460	1470	1480	1490	1500	1510	1520	1530	1540	1550	1560
HHTAGDFCER	CALGYYGIVK	GLPNDCQQCA	CPLISSSNNF	SPSCVAEGLD	DYRCTACPRG	YEGQYCERCA	PGYTGSPGNP	GGSCQECECD	PYGSLPVPCD	PVTGFCTCRP	GATGRKCDGC
1570	1580	1590	1600	1610	1620	1630	1640	1650	1660	1670	1680
KHWHAREGWE	CVFCGDECTG	LLLGDLARLE	QMVMSINLTG	PLPAPYKMLY	GLENMTQELK	HLLSPQRAPE	RLIQLAEGNL	NTLVTEMNEL	LTRATKVTAD	GEQTGQDAER	TNTRAKSLGE
1690	1700	1710	1720	1730	1740	1750	1760	1770	1780	1790	1800
FIKELARDAE	AVNEKAIKLN	ETLGTRDEAF	ERNLEGLQKE	IDQMIKELRR	KNLETQKEIA	EDELVAAEAL	LKKVKKLFGE	SRGENEEMEK	DLREKLADYK	NKVDDAWDLL	REATDKIREA
1810	1820	1830	1840	1850	1860	1870	1880	1890	1900	1910	1920
NRLFAVNQKN	MTALEKKKEA	VESGKRQIEN	TLKEGNDILD	EANRLADEIN	SIIDYVEDIQ	TKLPPMSEEL	NDKIDDLSQE	IKDRKLAEKV	SQAESHAAQL	NDSSAVLDGI	LDEAKNISFN
1930	1940	1950	1960	1970	1980	1990	2000	2010	2020	2030	2040
ATAAFKAYSN	IKDYIDEAEK	VAKEAKDLAH	EATKLATGPR	GLLKEDAKGC	LQKSFRILNE	AKKLANDVKE	NEDHLNGLKT	RIENADARNG	DLLRTLNDTL	GKLSAIPNDT	AAKLQAVKDK
2050	2060	2070	2080	2090	2100	2110	2120	2130	2140	2150	2160
ARQANDTAKD	VLAQITELHQ	NLDGLKKNYN	KLADSVAKTN	AVVKDPSKNK	IIADADATVK	NLEQEADRLI	DKLKPIKELE	DNLKKNISEI	KELINQARKQ	ANSIKVSVSS	GGDCIRTYKP
2170	2180	2190	2200	2210	2220	2230	2240	2250	2260	2270	2280

Protein Report

Cmpd.	No. of Cmpds.	m/z meas.	Δ m/z [ppm]	z	Rt [min]	Score	Site [%]	P	Range	Sequence	Modification	Type

Protein 9: alpha globin chain, partial [Homo sapiens]

Accession: gi|546234980

Database: NCBInr

Seq. Coverage [%]: 29.40 %

Score: 99.75

MW [kDa]: 5.70

pI: 7.78

No. of Peptides: 1

```
        10         20         30         40         50         60
MVLYPADKTN VKAAWGKVGA HAGEYGAEAL ERMFLSFPTT KTYFPHFDLS H
```

Cmpd.	No. of Cmpds.	m/z meas.	Δ m/z [ppm]	z	Rt [min]	Score	Site [%]	P	Range	Sequence	Modification	Type
16	2	1529.7405	4.05	1	14.20	99.75		0	18-32	K.VGAHAGEYGAEALER.M		CID

Protein 10: Oncostatin-M OS=Homo sapiens GN=OSM PE=1 SV=2

Accession: ONCM_HUMAN

Database: SwissProt

Seq. Coverage [%]: 3.20 %

Score: 80.81

MW [kDa]: 28.50

pI: 11.75

No. of Peptides: 2

Modification(s): Deamidated

```
        10         20         30         40         50         60         70         80         90        100        110        120
MGVLLTQRTL LSLVLALLFP SMASMAAIGS CSKEYRVLLG QLQKQTDLMQ DTSRLLDPYI RIQGLDVPKL REHCRERPGA FPSEETLRGL GRRGFLQTLN ATLGCVLHRL ADLEQRLPKA
       130        140        150        160        170        180        190        200        210        220        230        240
QDLERSGLNI EDLEKLQMAR PNILGLRNNI YCMAQLLDNS DTAEPTKAGR GASQPPTPTP ASDAFQRKLE GCRFLHGYHR FMHSVGRVFS KWGESPNRSR RHSPHQALRK GVRRTRPSRK
       250        260
GKRLMTRGQL PR
```

Protein Report

Cmpd.	No. of Cmpds.	m/z meas.	Δ m/z [ppm]	z	Rt [min]	Score	Site [%]	P	Range	Sequence	Modification	Type
67	2	870.5533	1200.94	1	17.35	36.69		0	62-69	R.IQGLDVPK.L		CID
37	1	870.5390	52.66	1	17.50	44.12		0	62-69	R.IQGLDVPK.L	Deamidated: 2	CID

Protein 11: unnamed protein product [Homo sapiens]

Accession: gi|194388808

Database: NCBInr

Seq. Coverage [%]: 4.70 %

Score: 76.03

MW [kDa]: 23.20

pI: 9.69

No. of Peptides: 1

10	20	30	40	50	60	70	80	90	100	110	120
MGKVKVGVNG	FGRIGRLVTR	AAFNSGKVDI	VAINDPFIDL	NYMVYMFQYD	STHGKFHGTV	KAENGKLVIN	GNPITIFQER	DPSKIKWGDA	GAEYVVESTG	VFTTMEKAGA	HLQGGAKRVI
130	140	150	160	170	180	190	200	210	220		
ISAPSADAPM	FVMGVNHEKY	DNSLKIISNA	SCTTNCLAPL	AKVIHDNFGI	VEGLMTTVHA	ITATLREAHW	HGLPCPHCQR	VSGGPDLPSR	KTCQI		

Cmpd.	No. of Cmpds.	m/z meas.	Δ m/z [ppm]	z	Rt [min]	Score	Site [%]	P	Range	Sequence	Modification	Type
17	1	1032.5970	2.07	1	14.95	76.03		1	4-13	K.VKVGVNGFGR.I		CID

Protein 12: Muellerian-inhibiting factor OS=Homo sapiens GN=AMH PE=1 SV=3

Accession: MIS_HUMAN

Database: SwissProt

Seq. Coverage [%]: 1.60 %

Score: 71.06

MW [kDa]: 59.20

pI: 7.93

No. of Peptides: 2

10	20	30	40	50	60	70	80	90	100	110	120
MRDLPLTSLA	LVLSALGALL	GTEALRAEEP	AVGTSGLIFR	EDLDWPPGSP	QEPLCLVALG	GDSNGSSSPL	RVVGALSAYE	QAFLGAVQRA	RWGPRDLATF	GVCNTGDRQA	ALPSLRRLGA
130	140	150	160	170	180	190	200	210	220	230	240
WLRDPGGQRL	VVLHLEEVTW	EPTPSLRFQE	PPPGGAGPPE	LALLVLYPGP	GPEVTVTRAG	LPGAQSLCPS	RDTRYLVLAV	DRPAGAWRGS	GLALTLQPRG	EDSRLSTARL	QALLFGDDHR
250	260	270	280	290	300	310	320	330	340	350	360
CFTRMTPALL	LLPRSEPAPL	PAHGQLDTVP	FPPPRPSAEL	EESPPSADPF	LETLTRLVRA	LRVPPARASA	PRLALDPDAL	AGFPQGLVNL	SDPAALERLL	DGEEPLLLLL	RPTAATTGDP
370	380	390	400	410	420	430	440	450	460	470	480
APLHDPTSAP	WATALARRVA	AELQAAAAEL	RSLPGLPPAT	APLLARLLAL	CPGGPGGLGD	PLRALLLLKA	LQGLRVEWRG	RDPRGPGRAQ	RSAGATAADG	PCALRELSVD	LRAERSVLIP
490	500	510	520	530	540	550	560	570			
ETYQANNCQG	VCGWPQSDRN	PRYGNHVVLL	LKMQVRGAAL	ARPPCCVPTA	YAGKLLISLS	EERISAHHVP	NMVATECGCR				

Cmpd.	No. of Cmpds.	m/z meas.	Δ m/z [ppm]	z	Rt [min]	Score	Site [%]	P	Range	Sequence	Modification	Type
81	2	856.5277	1195.84	1	17.35	32.45		0	109-116	R.QAALPSLR.R		CID
104	1	1011.6412	34.99	1	17.80	38.61		1	109-117	R.QAALPSLRR.L		CID

**Protein 13:**	PREDICTED: radial spoke head 1 homolog isoform X1 [Homo sapiens]		
**Accession:**	gi|530419415	**Score:**	66.66
**Database:**	NCBInr	**MW [kDa]:**	30.80
**Seq. Coverage [%]:**	4.80 %	**pI:**	4.27
		**No. of Peptides:**	1

10	20	30	40	50	60	70	80	90	100	110	120
MSDLGSEELE	EEGENDIGGI	YKFKNGARYI	GEYVRNKKHG	QGTFIYPDGS	RYEGEWANDL	RHGHGVYYYI	NNDTYTGEWF	AHQRHGQGTY	LYAETGSKYV	GTWVNGQQEG	TAELIHLNHR
130	140	150	160	170	180	190	200	210	220	230	240
YQGKFLNKNP	VGPGKYVFDV	GCEQHGEYRL	TDMERGEEEE	EEELVTVVPK	WKATQITELA	LWTPTLPKKP	TSTDGPGQDA	PGAESAGEPG	EEAQALLEGF	EGEMDMRPGD	EDADVLREES
250	260	270	280								
REYDQEEFRY	DMDEGNINSE	EEETRQSDLQ	D								

Protein Report

Cmpd.	No. of Cmpds.	m/z meas.	Δ m/z [ppm]	z	Rt [min]	Score	Site [%]	P	Range	Sequence	Modification	Type
19	1	1434.6867	7.39	1	14.95	66.66		0	39-51	K.HGQGTFIYPDGSR.Y		CID

**Protein 14:**	Phosphatidylinositol 3,4,5-trisphosphate-dependent Rac exchanger 2 protein OS=Homo sapiens GN=PREX2 PE=2 SV=1		
**Accession:**	PREX2_HUMAN	**Score:**	64.74
**Database:**	SwissProt	**MW [kDa]:**	182.50
**Seq. Coverage [%]:**	1.40 %	**pI:**	7.60
		**No. of Peptides:**	2
**Modification(s):**	Carbamyl, Oxidation, Deamidated		

Protein Report

10	20	30	40	50	60	70	80	90	100	110	120
MSEDSRGDSR	AESAKDLEKQ	LRLRVCVLSE	LQKTERDYVG	TLEFLVSAFL	HRMNQCAASK	VDKNVTEETV	KMLFSNIEDI	LAVHKEFLKV	VEECLHPEPN	AQQEVGTCFL	HFKDKFRIYD
130	140	150	160	170	180	190	200	210	220	230	240
EYCSNHEKAQ	KLLLELNKIR	TIRTFLLNCM	LLGGRKNTDV	PLEGYLVTPI	QRICKYPLIL	KELLKRTPRK	HSDYAAVMEA	LQAMKAVCSN	INEAKRQMEK	LEVLEEWQSH	IEGWEGSNIT
250	260	270	280	290	300	310	320	330	340	350	360
DTCTEMLMCG	VLLKISSGNI	QERVFFLFDN	LLVYCKRKHR	RLKNSKASTD	GHRYLFRGRI	NTEVMEVENV	DDGTADFHSS	GHIVVNGWKI	HNTAKNKWFV	CMAKTPEEKH	EWFEAILKER
370	380	390	400	410	420	430	440	450	460	470	480
ERRKGLKLGM	EQDTWVMISE	QGEKLYKMMC	RQGNLIKDRK	RKLTTFPKCF	LGSEFVSWLL	EIGEIHRPEE	GVHLGQALLE	NGIIHHVTDK	HQFKPEQMLY	RFRYDDGTFY	PRNEMQDVIS
490	500	510	520	530	540	550	560	570	580	590	600
KGVRLYCRLH	SLFTPVIRDK	DYHLRTYKSV	VMANKLIDWL	IAQGDCRTRE	EAMIFGVGLC	DNGFMHHVLE	KSEFKDEPLL	FRFFSDEEME	GSNMKHRLMK	HDLKVVENVI	AKSLLIKSNE
610	620	630	640	650	660	670	680	690	700	710	720
GSYGFGLEDK	NKVPIIKLVE	KGSNAEMAGM	EVGKKIFAIN	GDLVFMRPFN	EVDCFLKSCL	NSRKPLRVLV	STKPRETVKI	PDSADGLGFQ	IRGFGPSVVH	AVGRGTVAAA	AGLHPGQCII
730	740	750	760	770	780	790	800	810	820	830	840
KVNGINVSKE	THASVIAHVT	ACRKYRRPTK	QDSIQWVYNS	IESAQEDLQK	SHSKPPGDEA	GDAFDCKVEE	VIDKFNTMAI	IDGKKEHVSL	TVDNVHLEYG	VVYEYDSTAG	IKCNVVEKMI
850	860	870	880	890	900	910	920	930	940	950	960
EPKGFFSLTA	KILEALAKSD	EHFVQNCTSL	NSLNEVIPTD	LQSKFSALCS	ERIEHLCQRI	SSYKKFSRVL	KNRAWPTFKQ	AKSKISPLHS	SDFCPTNCHV	NVMEVSYPKT	STSLGSAFGV
970	980	990	1000	1010	1020	1030	1040	1050	1060	1070	1080
QLDSRKHNSH	DKENKSSEQG	KLSPMVYIQH	TITTMAAPSG	LSLGQQDGHG	LRYLLKEEDL	ETQDIYQKLL	GKLQTALKEV	EMCVCQIDDL	LSSITYSPKL	ERKTSEGIIP	TDSDNEKGER
1090	1100	1110	1120	1130	1140	1150	1160	1170	1180	1190	1200
NSKRVCFNVA	GDEQEDSGHD	TISNRDSYSD	CNSNRNSIAS	FTSICSSQCS	SYFHSDEMDS	GDELPLSVRI	SHDKQDKIHS	CLEHLFSQVD	SITNLLKGQA	VVRAFDQTKY	LTPGRGLQEF
1210	1220	1230	1240	1250	1260	1270	1280	1290	1300	1310	1320
QQEMEPKLSC	PKRLRLHIKQ	DPWNLPSSVR	TLAQNIRKFV	EEVKCRLLLA	LLEYSDSETQ	LRRDMVFCQT	LVATVCAFSE	QLMAALNQMF	DNSKENEMET	WEASRRWLDQ	IANAGVLFHF
1330	1340	1350	1360	1370	1380	1390	1400	1410	1420	1430	1440
QSLLSPNLTD	EQAMLEDTLV	ALFDLEKVSF	YFKPSEEEPL	VANVPLTYQA	EGSRQALKVY	FYIDSYHFEQ	LPQRLKNGGG	FKIHPVLFAQ	ALESMEGYYY	RDNVSVEEFQ	AQINAASLEK
1450	1460	1470	1480	1490	1500	1510	1520	1530	1540	1550	1560
VKQYNQKLRA	FYLDKSNSPP	NSTSKAAYVD	KLMRPLNALD	ELYRLVASFI	RSKRTAACAN	TACSASGVGL	LSVSSELCNR	LGACHIIMCS	SGVHRCTLSV	TLEQAIILAR	SHGLPPRYIM
1570	1580	1590	1600	1610							
QATDVMRKQG	ARVQNTAKNL	GVRDRTPQSA	PRLYKLCEPP	PPAGEE							

Cmpd.	No. of Cmpds.	m/z meas.	Δ m/z [ppm]	z	Rt [min]	Score	Site [%]	P	Range	Sequence	Modification	Type
151	1	1724.8605	537.10	1	30.55	38.87		1	141-155	R.TIRTFLLNCMLLGGR.K	Oxidation: 10	CID
52	1	993.4991	-12.21	1	15.70	25.87		1	1443-1449	K.QYNQKLR.A	Carbamyl: 5; Deamidated: 1	CID

Protein Report

**Protein 15:**	MAX gene-associated protein OS=Homo sapiens GN=MGA PE=1 SV=3		
**Accession:**	MGAP_HUMAN	**Score:**	57.27
**Database:**	SwissProt	**MW [kDa]:**	331.60
**Seq. Coverage [%]:**	0.80 %	**pI:**	6.35
		**No. of Peptides:**	2

Protein Report

10	20	30	40	50	60	70	80	90	100	110	120
MEEKQQIILA	NQDGGTVAGA	APTFFVILKQ	PGNGKTDQGI	LVTNQDACAL	ASSVSSPVKS	KGKICLPADC	TVGGITVTLD	NNSMWNEFYH	RSTEMILTKQ	GRRMFPYCRY	WITGLDSNLK
130	140	150	160	170	180	190	200	210	220	230	240
YILVMDISPV	DNHRYKWNGR	WWEPSGKAEP	HVLGRVFIHP	ESPSTGHYWM	HQPVSFYKLK	LTNNTLDQEG	HIILHSMHRY	LPRLHLVPAE	KAVEVIQLNG	PGVHTFTFPQ	TEFFAVTAYQ
250	260	270	280	290	300	310	320	330	340	350	360
NIQITQLKID	YNPFAKGFRD	DGLNNKPQRD	GKQKNSSDQE	GNNISSSSGH	RVRLTEGQGS	EIQPGDLDPL	SRGHETSGKG	LEKTSLNIKR	DFLGFMDTDS	ALSEVPQLKQ	EISECLIASS
370	380	390	400	410	420	430	440	450	460	470	480
FEDDSRVASP	LDQNGSFNVV	IKEEPLDDYD	YELGECPEGV	TVKQEETDEE	TDVYSNSDDD	PILEKQLKRH	NKVDNPEADH	LSSKWLPSSP	SGVAKAKMFK	LDTGKMPVVY	LEPCAVTRST
490	500	510	520	530	540	550	560	570	580	590	600
VKISELPDNM	LSTSRKDKSS	MLAELEYLPT	YIENSNETAF	CLGKESENGL	RKHSPDLRVV	QKYPLLKEPQ	WKYPDISDSI	STERILDDSK	DSVGDSLSGK	EDLGRKRTTM	LKIATAAKVV
610	620	630	640	650	660	670	680	690	700	710	720
NANQNASPNV	PGKRGRPRKL	KLCKAGRPPK	NTGKSLISTK	NTPVSPGSTF	PDVKPDLEDV	DGVLFVSFES	KEALDIHAVD	GTTEESSSLQ	ASTTNDSGYR	ARISQLEKEL	IEDLKTLRHK
730	740	750	760	770	780	790	800	810	820	830	840
QVIHPGLQEV	GLKLNSVDPT	MSIDLKYLGV	QLPLAPATSF	PFWNLTGTNP	ASPDAGFPFV	SRTGKTNDFT	KIKGWRGKFH	SASASRNEGG	NSESSLKNRS	AFCSDKLDEY	LENEGKLMET
850	860	870	880	890	900	910	920	930	940	950	960
SMGFSSNAPT	SPVVYQLPTK	STSYVRTLDS	VLKKQSTISP	STSYSLKPHS	VPPVSRKAKS	QNRQATFSGR	TKSSYKSILP	YPVSPKQKYS	HVILGDKVTK	NSSGIISENQ	ANNFVVPTLD
970	980	990	1000	1010	1020	1030	1040	1050	1060	1070	1080
ENIFPKQISL	RQAQQQQQQQ	QGSRPPGLSK	SQVKLMDLED	CALWEGKPRT	YITEERADVS	LTTLLTAQAS	LKTKPIHTII	RKRAPPCNND	FCRLGCVCSS	LALEKRQPAH	CRRPDCMFGC
1090	1100	1110	1120	1130	1140	1150	1160	1170	1180	1190	1200
TCLKRKVVLV	KGGSKTKHFQ	RKAAHRDPVF	YDTLGEEARE	EEEGIREEEE	QLKEKKKRKK	LEYTICETEP	EQPVRHYPLW	VKVEGEVDPE	PVYIPTPSVI	EPMKPLLLPQ	PEVLSPTVKG
1210	1220	1230	1240	1250	1260	1270	1280	1290	1300	1310	1320
KLLTGIKSPR	SYTPKPNPVI	REEDKDPVYL	YFESMMTCAR	VRVYERKKED	QRQPSSSSSP	SPSFQQQTSC	HSSPENHNNA	KEPDSEQQPL	KQLTCDLEDD	SDKLQEKSWK	SSCNEGESSS
1330	1340	1350	1360	1370	1380	1390	1400	1410	1420	1430	1440
TSYMHQRSPG	GPTKLIEIIS	DCNWEEDRNK	ILSILSQHIN	SNMPQSLKVG	SFIIELASQR	KSRGEKNPPV	YSSRVKISMP	SCQDQDDMAE	KSGSETPDGP	LSPGKMEDIS	PVQTDALDSV
1450	1460	1470	1480	1490	1500	1510	1520	1530	1540	1550	1560
RERLHGGKGL	PFYAGLSPAG	KLVAYKRKPS	SSTSGLIQVA	SNAKVAASRK	PRTLLPSTSN	SKMASSSGTA	TNRPGKNLKA	FVPAKRPIAA	RPSPGGVFTQ	FVMSKVGALQ	QKIPGVSTPQ
1570	1580	1590	1600	1610	1620	1630	1640	1650	1660	1670	1680
TLAGTQKFSI	RPSPVMVVTP	VVSSEPVQVC	SPVTAAVTTT	TPQVFLENTT	AVTPMTAISD	VETKETTYSS	GATTTGVVEV	SETNTSTSVT	STQSTATVNL	TKTTGITTPV	ASVAFPKSLV
1690	1700	1710	1720	1730	1740	1750	1760	1770	1780	1790	1800
ASPSTITLPV	ASTASTSLVV	VTAAASSSMV	TTPTSSLGSV	PIILSGINGS	PPVSQRPENA	AQIPVATPQV	SPNTVKRAGP	RLLHPNGQIV	QLLPLHQLRG	SNTQPNLQPV	MFRNPGSVMG
1810	1820	1830	1840	1850	1860	1870	1880	1890	1900	1910	1920
IRLPAPSKPS	ETPPSSTSSS	AFSVMNPVIQ	AVGSSSAVNV	ITQAPSLLSS	GASFVSQAGT	LTLRISPPEP	QSFASKTGSE	TKITYSSGGQ	PVGTASLIPL	QSGSFALLQL	PGQKPVPSSI
1930	1940	1950	1960	1970	1980	1990	2000	2010	2020	2030	2040
LQHVASLQMK	RESQNPDQKD	ETNSIKREQE	TKKVLQSEGE	AVDPEANVIK	QNSGAATSEE	TLNDSLEDRG	DHLDEECLPE	EGCATVKPSE	HSCITGSHTD	QDYKDVNEEY	GARNRKSSKE
2050	2060	2070	2080	2090	2100	2110	2120	2130	2140	2150	2160

Cmpd.	No. of Cmpds.	m/z meas.	Δ m/z [ppm]	z	Rt [min]	Score	Site [%]	P	Range	Sequence	Modification	Type
12	1	1833.8061	-71.63	1	12.85	20.30		2	483-498	K.ISELPDNMLSTSRKDK.S		CID
54	1	1027.5137	-66.69	1	16.90	36.97		1	2474-2481	K.KLEYIYAK.Q		CID

**Protein 16:**	hCG2039963 [Homo sapiens]		
**Accession:**	gi|119606679	**Score:**	54.57
**Database:**	NCBInr	**MW [kDa]:**	7.90
**Seq. Coverage [%]:**	22.50 %	**pI:**	10.79
		**No. of Peptides:**	1
**Modification(s):**	Carbamidomethyl, Carboxymethyl, Deamidated		

```
        10        20        30        40        50        60        70        80        90
AGAFGCSVAT RGPGGLTRQQ DSCMGIGDHG SAAGARLSSR PADDGVSLCR AGWRAMVPSW ITAISNLCFP GSSDSPASSS
```

Cmpd.	No. of Cmpds.	m/z meas.	Δ m/z [ppm]	z	Rt [min]	Score	Site [%]	P	Range	Sequence	Modification	Type
124	1	1819.8843	624.03	1	22.75	54.57		0	19-36	R.QQDSCMGIGDHGSAAGAR.L	Carbamidomethyl: 5; Deamidated: 2	CID

**Protein 17:**	PREDICTED: nuclear respiratory factor 1 isoform X3 [Homo sapiens]		
**Accession:**	gi|530385993	**Score:**	52.48
**Database:**	NCBInr	**MW [kDa]:**	51.30
**Seq. Coverage [%]:**	2.50 %	**pI:**	5.18
		**No. of Peptides:**	1
**Modification(s):**	Deamidated		

Protein Report

10	20	30	40	50	60	70	80	90	100	110	120
MEEHGVTQTE	HMATIEAHAV	AQQVQQVHVA	TYTEHSMLSA	DEDSPSSPED	TSYDDSDILN	STAADEVTAH	LAAAGPVGMA	AAAAVATGKK	RKRPHVFESN	PSIRKRQQTR	LLRKLRATLD
**130**	**140**	**150**	**160**	**170**	**180**	**190**	**200**	**210**	**220**	**230**	**240**
EYTTRVGQQA	IVLCISPSKP	NPVFKVFGAA	PLENVVRKYK	SMILEDLESA	LAEHAPAPQE	VNSELPPLTI	DGIPVSVDKM	TQAQLRAFIP	EMLKYSTGRG	KPGWGKESCK	PIWWPEDIPW
**250**	**260**	**270**	**280**	**290**	**300**	**310**	**320**	**330**	**340**	**350**	**360**
ANVRSDVRTE	EQKQRVSWTQ	ALRTIVKNCY	KQHGREDLLY	AFEDQQTQTQ	ATATHSIAHL	VPSQTVVQTF	SNPDGTVSLI	QVGTGATVAT	LADASELPTT	VTVAQVNYSA	VADGEVEQNW
**370**	**380**	**390**	**400**	**410**	**420**	**430**	**440**	**450**	**460**	**470**	**480**
ATLQGGEMTI	QTTQASEATQ	AVASLAEAAV	AASQEMQQGA	TVTMALNSEA	AAHAVATLAE	ATLQGGGQIV	LSGETAAAVG	ALTGVQDANA	PHRVFHLALP	TALAWGPPEI	LKSTSVSAGQ
**490**											
H											

Cmpd.	No. of Cmpds.	m/z meas.	Δ m/z [ppm]	z	Rt [min]	Score	Site [%]	P	Range	Sequence	Modification	Type
71	1	1439.8188	739.44	1	30.85	52.48		0	93-104	K.RPHVFESNPSIR.K		CID

Protein 18: Fibronectin type III domain-containing protein 1 OS=Homo sapiens GN=FNDC1 PE=1 SV=4

Accession: FNDC1_HUMAN

Database: SwissProt

Seq. Coverage [%]: 0.90 %

Score: 50.64

MW [kDa]: 205.40

pI: 9.79

No. of Peptides: 0

Protein Report

10	20	30	40	50	60	70	80	90	100	110	120
MAPEAGATLR	APRRLSWAAL	LLLAALLPVA	SSAAASVDHP	LKPRHVKLLS	TKMGLKVTWD	PPKDATSRPV	EHYNIAYGKS	LKSLKYIKVN	AETYSFLIED	VEPGVVYFVL	LTAENHSGVS
**130**	**140**	**150**	**160**	**170**	**180**	**190**	**200**	**210**	**220**	**230**	**240**
RPVYRAESPP	GGEWIEIDGF	PIKGPGPFNE	TVTEKEVPNK	PLRVRVRSSD	DRLSVAWKAP	RLSGAKSPRR	SRGFLLGYGE	SGRKMNYVPL	TRDERTHEIK	KLASESVYVV	SLQSMNSQGR
**250**	**260**	**270**	**280**	**290**	**300**	**310**	**320**	**330**	**340**	**350**	**360**
SQPVYRAALT	KRKISEEDEL	DVPDDISVRV	MSSQSVLVSW	VDPVLEKQKK	VVASRQYTVR	YREKGELARW	DYKQIANRRV	LIENLIPDTV	YEFAVRISQG	ERDGKWSTSV	FQRTPESAPT
**370**	**380**	**390**	**400**	**410**	**420**	**430**	**440**	**450**	**460**	**470**	**480**
TAPENLNVWP	VNGKPTVVAA	SWDALPETEG	KVKEYILSYA	PALKPFGAKS	LTYPGDTTSA	LVDGLQPGER	YLFKIRATNR	RGLGPHSKAF	IVAMPTTSKA	DVEQNTEDNG	KPEKPEPSSP
**490**	**500**	**510**	**520**	**530**	**540**	**550**	**560**	**570**	**580**	**590**	**600**
SPRAPASSQH	PSVPASPQGR	NAKDLLLDLK	NKILANGGAP	RKPQLRAKKA	EELDLQSTEI	TGEEELGSRE	DSPMSPSDTQ	DQKRTLRPPS	RHGHSVVAPG	RTAVRARMPA	LPRREGVDKP
**610**	**620**	**630**	**640**	**650**	**660**	**670**	**680**	**690**	**700**	**710**	**720**
GFSLATQPRP	GAPPSASASP	AHHASTQGTS	HRPSLPASLN	DNDLVDSDED	ERAVGSLHPK	GAFAQPRPAL	SPSRQSPSSV	LRDRSSVHPG	AKPASPARRT	PHSGAAEEDS	SASAPPSRLS
**730**	**740**	**750**	**760**	**770**	**780**	**790**	**800**	**810**	**820**	**830**	**840**
PPHGGSSRLL	PTQPHLSSPL	SKGGKDGEDA	PATNSNAPSR	STMSSSVSSH	LSSRTQVSEG	AEASDGESHG	DGDREDGGRQ	AEATAQTLRA	RPASGHFHLL	RHKPFAANGR	SPSRFSIGRG
**850**	**860**	**870**	**880**	**890**	**900**	**910**	**920**	**930**	**940**	**950**	**960**
PRLQPSSSPQ	STVPSRAHPR	VPSHSDSHPK	LSSGIHGDEE	DEKPLPATVV	NDHVPSSSRQ	PISRGWEDLR	RSPQRGASLH	RKEPIPENPK	STGADTHPQG	KYSSLASKAQ	DVQQSTDADT
**970**	**980**	**990**	**1000**	**1010**	**1020**	**1030**	**1040**	**1050**	**1060**	**1070**	**1080**
EGHSPKAQPG	STDRHASPAR	PPAARSQQHP	SVPRRMTPGR	APQQQPPPPV	ATSQHHPGPQ	SRDAGRSPSQ	PRLSLTQAGR	PRPTSQGRSH	SSSDPYTASS	RGMLPTALQN	QDEDAQGSYD
**1090**	**1100**	**1110**	**1120**	**1130**	**1140**	**1150**	**1160**	**1170**	**1180**	**1190**	**1200**
DDSTEVEAQD	VRAPAHAARA	KEAAASLPKH	QQVESPTGAG	AGGDHRSQRG	HAASPARPSR	PGGPQSRARV	PSRAAPGKSE	PPSKRPLSSK	SQQSVSAEDD	EEEDAGFFKG	GKEDLLSSSV
**1210**	**1220**	**1230**	**1240**	**1250**	**1260**	**1270**	**1280**	**1290**	**1300**	**1310**	**1320**
PKWPSSSTPR	GGKDADGSLA	KEEREPAIAL	APRGGSLAPV	KRPLPPPPGS	SPRASHVPSR	LPPRSAATVS	PVAGTHPWPQ	YTTRAPPGHF	STTPMLSLRQ	RMMHARFRNP	LSRQPARPSY
**1330**	**1340**	**1350**	**1360**	**1370**	**1380**	**1390**	**1400**	**1410**	**1420**	**1430**	**1440**
RQGYNGRPNV	EGKVLPGSNG	KPNGQRIING	PQGTKWVVDL	DRGLVLNAEG	RYLQDSHGNP	LRIKLGGDGR	TIVDLEGTPV	VSPDGLPLFG	QGRHGTPLAN	AQDKPILSLG	GKPLVGLEVI
**1450**	**1460**	**1470**	**1480**	**1490**	**1500**	**1510**	**1520**	**1530**	**1540**	**1550**	**1560**
KKTTHPPTTT	MQPTTTTTPL	PTTTTPRPTT	ATTRRTTTTR	RTTTRRPTTT	VRTTTRTTTT	TTPTPTTPIP	TCPPGTLERH	DDDGNLIMSS	NGIPECYAEE	DEFSGLETDT	AVPTEEAYVI
**1570**	**1580**	**1590**	**1600**	**1610**	**1620**	**1630**	**1640**	**1650**	**1660**	**1670**	**1680**
YDEDYEFETS	RPPTTTEPST	TATTPRVIPE	EGAISSFPEE	EFDLAGRKRF	VAPYVTYLNK	DPSAPCSLTD	ALDHFQVDSL	DEIIPNDLKK	SDLPPQHAPR	NITVVAVEGC	HSFVIVDWDK
**1690**	**1700**	**1710**	**1720**	**1730**	**1740**	**1750**	**1760**	**1770**	**1780**	**1790**	**1800**
ATPGDVVTGY	LVYSASYEDF	IRNKWSTQAS	SVTHLPIENL	KPNTRYYFKV	QAQNPHGYGP	ISPSVSFVTE	SDNPLLVVRP	PGGEPIWIPF	AFKHDPSYTD	CHGRQYVKRT	WYRKFVGVVL
**1810**	**1820**	**1830**	**1840**	**1850**	**1860**	**1870**	**1880**	**1890**	**1900**		
CNSLRYKIYL	SDNLKDTFYS	IGDSWGRGED	HCQFVDSHLD	GRTGPQSYVE	ALPTIQGYYR	QYRQEPVRFG	NIGFGTPYYY	VGWYECGVSI	PGKW		

Cmpd.	No. of Cmpds.	m/z meas.	Δ m/z [ppm]	z	Rt [min]	Score	Site [%]	P	Range	Sequence	Modification	Type

Protein 19: COL1A1 and PDGFB fusion transcript [Homo sapiens]

**Accession:**	gi|3288487	**Score:**	50.41
**Database:**	NCBInr	**MW [kDa]:**	37.00
**Seq. Coverage [%]:**	5.10 %	**pI:**	5.09
		**No. of Peptides:**	1

10	20	30	40	50	60	70	80	90	100	110	120
ALLVPLELVV	PPETVVSLVP	PALLALLAPL	VLTANLVLKA	NLVMLVPKAM	LVPLGLPDPL	DPLAPLVMLV	LLEPKVLAAA	LVPLVLLVSL	VLLAESVLLA	PLEMLDPLAL	LVLLAKKAAK
130	140	150	160	170	180	190	200	210	220	230	240
VPVVRLALLD	VLVKLVPLVP	LALLARKDPL	VLMVLLVLLV	LPGLKVLLDS	VVWSACLVRE	EREASLVFLA	PLVNLANKVP	LEQVVNVVPP	VPWAPLDWLD	PLVNLDVRGL	LLPKVPLDET
250	260	270	280	290	300	310	320	330	340	350	360
VLLAPRVTVV	RPAPLDPLVL	WCSGAPGPVG	PAGKSGDRGE	TGPAGPAGPV	GPVGARGPAG	PQGPRGDKGE	TGEQGDRGIK	GHRGFSGLQG	PPGPPGDPIP	EELYEMLSDH	SIRSF

Cmpd.	No. of Cmpds.	m/z meas.	Δ m/z [ppm]	z	Rt [min]	Score	Site [%]	P	Range	Sequence	Modification	Type
114	1	1546.8081	7.02	1	17.80	50.41		0	279-296	R.GETGPAGPAGPVGPVGAR.G		CID

Protein 20: unnamed protein product [Homo sapiens]

**Accession:**	gi|7022948	**Score:**	47.85
**Database:**	NCBInr	**MW [kDa]:**	49.00
**Seq. Coverage [%]:**	4.00 %	**pI:**	9.90
		**No. of Peptides:**	1

10	20	30	40	50	60	70	80	90	100	110	120
MKNALIPRIK	NACLQTSSLA	VRVNSLVCLG	KILEYLDKWF	VLDDILPFLQ	QIPSKEPAVL	MGILGIYKCT	FTHKKLGITK	EQLAGKVLPH	LIPLSIENNL	NLNQFNSFIS	VIKEMLNRLE
130	140	150	160	170	180	190	200	210	220	230	240
SEHKTKLEQL	HIMQEQQKSL	DIGNQMNVSE	EMKVTNIGNQ	QIDKVFNNIG	ADLLTGSESE	NKEDGLQNKH	KRASLTLEEK	QKLAKEQEQA	QKLKSQQPLK	PQVHTPVATV	KQTKDLTDTL
250	260	270	280	290	300	310	320	330	340	350	360
MDNMSSLTSL	SVSTPKSSAS	STFASVPSMG	IGMMFSTPTD	NTKRNLTNGL	NANMGFQTSG	FNMPVNTNQN	FYSSPSTVGV	TKMTLGTPPT	LPNFNALSVP	PAGAKQTQQR	PTDMSALNNL
370	380	390	400	410	420	430	440	450			
FGPQKPKVSM	NQLSQQKPNQ	WLNQFVPPQG	SPTMGSSVMG	TQMNVIGQSA	FGMQGNPFFN	PQNFAQPPTT	MTNSSSASND	LKDLFG			

Cmpd.	No. of Cmpds.	m/z meas.	Δ m/z [ppm]	z	Rt [min]	Score	Site [%]	P	Range	Sequence	Modification	Type
75	1	1907.9394	2.55	1	29.95	47.85		0	165-182	K.VFNNIGADLLTGSESENK.E		CID

Protein 21: Tetratricopeptide repeat protein 34 OS=Homo sapiens GN=TTC34 PE=2 SV=2

Accession: TTC34_HUMAN | **Score:** 46.12

Database: SwissProt | **MW [kDa]:** 60.90

Seq. Coverage [%]: 1.60 % | **pI:** 7.22

No. of Peptides: 0

10	20	30	40	50	60	70	80	90	100	110	120
MLQRSPRAGP	SRAQGRREAA	ETGGPTTQEG	VACGVHQLAT	LLMELDSEDE	ASRLLAADAL	YRLGRLEETH	KALLVALSRR	PQAAPVLARL	ALLQLRRGFF	YDANQLVKKL	VQSGDTACLQ
130	140	150	160	170	180	190	200	210	220	230	240
PTLDVFCHED	RQLLQGHCHA	RALAILRARP	GGADGRVHTK	EAIAYLSLAI	FAAGSQASES	LLARARCYGF	LGQKKTAMFD	FNTVLRAEPG	NVQALCGRAL	VHLALDQLQE	AVDDIVSALK
250	260	270	280	290	300	310	320	330	340	350	360
LGPGTVVPEL	RSLKPEAQAL	ITQGLYSHCR	ALLSQLPDTG	APLEDKDTQG	LLAVGEALIK	IDSGQPHWHL	LLADILMAQG	SYEEAGTHLE	KALHRAPTSE	AARARLGLLQ	LKKGDVPGAA
370	380	390	400	410	420	430	440	450	460	470	480
RDLQSLAEVD	APDLSCLLHL	LEASERQSLA	QAAAQEAGTL	LDAGQPRQAL	GYCSLSVLAS	GSSACHLRLR	ATCLAELQEF	GRALRDLDHV	LQEALGDGDL	PRRAEDFCRQ	GRLLLSLGDE
490	500	510	520	530	540	550	560	570			
AAAAGAFAQA	LKLAPSLAQN	SLCRQPGRAP	TARMFLLRGQ	CCLEEQRHAE	AWTAVESGLL	VDPDHRGLKR	LKARIRREAS	SGCWLQ			

Cmpd.	No. of Cmpds.	m/z meas.	Δ m/z [ppm]	z	Rt [min]	Score	Site [%]	P	Range	Sequence	Modification	Type

Protein 22: fibronectin type III domain containing 3B, isoform CRA_d [Homo sapiens]

Accession: gi|119598879 | **Score:** 45.53

Database: NCBInr | **MW [kDa]:** 45.30

Seq. Coverage [%]: 7.40 % | **pI:** 8.61

No. of Peptides: 1

Modification(s): Carbamyl, Deamidated

Protein Report

10	20	30	40	50	60	70	80	90	100	110	120
MYVTMMMTDQ	IPLELPPLLN	GEVAMMPHLV	NGDAAQQVIL	VQVNPGETFT	IRAEDGTLQC	IQGPAEVPMM	SPNGSIPPIH	VPPGYISQVI	EDSTGVRRVV	VTPQSPECYP	PSYPSAMSPT
**130**	**140**	**150**	**160**	**170**	**180**	**190**	**200**	**210**	**220**	**230**	**240**
HHLPPYLTHH	PHFIHNSHTA	YYPPVTGPGD	MPPQFFPQHH	LPHTIYGEQE	IIPFYGMSSY	ITREDQYSKP	PHKKLKDRQI	DRQNRLNSPP	SSIYKSSCTT	VYNGYGKGHS	GGSGGGGSGS
**250**	**260**	**270**	**280**	**290**	**300**	**310**	**320**	**330**	**340**	**350**	**360**
GPGIKKTERR	ARSSPKSNDS	DLQEYELEVK	RVQDILSGIE	KPQVRTRPTD	KSKSMILTPV	YAMYNSVKGS	CSEPVSFTTH	SCAPECPFPP	KLAHRSKSSL	TLQWKAPIDN	GSKITNYLLE
**370**	**380**	**390**	**400**	**410**							
WDENVLYRMP	PLLFMSPEAL	GRYLRWSVLI	LKATVCQLVD	PVHFWPS							

Cmpd.	No. of Cmpds.	m/z meas.	Δ m/z [ppm]	z	Rt [min]	Score	Site [%]	P	Range	Sequence	Modification	Type
67	1	2717.0836	-37.53	1	16.45	45.53		1	216-245	K.SSCTTVYNGYGKGHSGGSGGGGSGSGPGIK.K	Carbamyl: 12; Deamidated: 8	CID

Protein 23: unnamed protein product [Homo sapiens]

Accession: gi|47077801

Database: NCBInr

Seq. Coverage [%]: 1.90 %

Score: 41.19

MW [kDa]: 84.40

pI: 6.41

No. of Peptides: 1

Protein Report

10	20	30	40	50	60	70	80	90	100	110	120
MAYHEKPVLP	PPLIILSHIV	SLFCCICKRR	KKDKTSDGPK	LFLTEEDQKK	LHDFEEQCVE	MYFNEKDDKF	HSGSEERIRV	TFERVEQMCI	QIKEVGDRVN	YIKRSLQSLD	SQIGHLQDLS
130	140	150	160	170	180	190	200	210	220	230	240
ALTVDTLKTL	TAQKASEASK	VHNEITRELS	ISKHLAQNLI	DDGPVRPSVW	KKHGVVNTLS	SSLPQGDLES	NNPFHCNILM	KDDKDPQCNI	FGQDLPAVPQ	RKEFNFPEAG	SSSGALFPSA
250	260	270	280	290	300	310	320	330	340	350	360
VSPPELRQRL	HGVELLKIFN	KNQKLGSSST	SIPHLSSPPT	KFFVSTPSQP	SCKSHLETGT	KDQETVCSKA	TEGDNTEFGA	FVGHRDSMDL	QRFKETSNKI	KILSNNNTSE	NTLKRVSSLA
370	380	390	400	410	420	430	440	450	460	470	480
GFIDCHRTSI	PVHSKQAEKI	SRRPSTEDTH	EVDSKAALIP	DWLQDRPSNR	EMPSEEGTLN	GLTSPFKPAM	DTNYYYSAVE	RNNLMRLSQS	IPFTPVPPRG	EPVTVYRLEE	SSPNILNNSM
490	500	510	520	530	540	550	560	570	580	590	600
SSWSQLGLCA	KIEFLSKEEM	GGGLRRAVKV	QCTWSEHDIL	KSGHLYIIKS	FLPEVVNTWS	SIYKEDTVLH	LCLREIQQQR	AAQKLTFAFN	QMKPKSIPYS	PRFLEVFLLY	CHSAGQWFAV
610	620	630	640	650	660	670	680	690	700	710	720
EECMTGEFRK	YNNNNGDEII	PTNTLEEIML	AFSHWTYEYT	RGELLVLDLQ	GVGENLTDPS	VIKAEEKRSC	DMVFGPANLG	EDAIKNFRAK	HHCNSCCRKL	KLPDLKRNDY	TPDKIIFPQD
730	740	750									
EPSDLNLQPG	NSTKESESTN	SVRLML									

Cmpd.	No. of Cmpds.	m/z meas.	Δ m/z [ppm]	z	Rt [min]	Score	Site [%]	P	Range	Sequence	Modification	Type
80	1	1567.7542	1248.55	1	30.55	41.19		1	492-505	K.IEFLSKEEMGGGLR.R		CID

Protein 24: unnamed protein product [Homo sapiens]

Accession: gi|14041882

Database: NCBInr

Seq. Coverage [%]: 5.40 %

Score: 38.59

MW [kDa]: 19.50

pI: 11.90

No. of Peptides: 0

Modification(s): Propionamide

10	20	30	40	50	60	70	80	90	100	110	120
MGAGGRRMRG	APARLLLPLL	PWLLLLLAPE	ARGAPGCPLS	IRSCKCSGER	PKGLSGGVPG	PARRRVVCSG	GDLPEPPEPG	LLPNGTVTLL	LSNNKITGLR	NGSFLGLSLL	EKLDLRNNII
130	140	150	160	170	180	190					
STVQPGAFLG	LGELKRLDLS	NNRIGCLTSE	TFQGLPRLLR	LNISGNIFSS	LQPGVFDELP	ALKVV					

Cmpd.	No. of Cmpds.	m/z meas.	Δ m/z [ppm]	z	Rt [min]	Score	Site [%]	P	Range	Sequence	Modification	Type

Protein 25: tRNA wybutosine-synthesizing protein 1 homolog B OS=Homo sapiens GN=TYW1B PE=2 SV=2

**Accession:**	TYW1B_HUMAN	**Score:**	36.05
**Database:**	SwissProt	**MW [kDa]:**	76.90
**Seq. Coverage [%]:**	1.30 %	**pI:**	5.84
		**No. of Peptides:**	1

Modification(s): Oxidation

10	20	30	40	50	60	70	80	90	100	110	120
MDPSADTWDL	SSPLISLWIN	RFYIYLGFAV	SISLWICVQI	VIEMQGFATV	LAEAVTSLDL	PVAIINLKEY	DPDDHLIEEV	TSKNVCVFLV	ATYTDGLPTE	SAEWFCKWLE	EASIDFRFGK
130	140	150	160	170	180	190	200	210	220	230	240
TYLKGMRDAV	FGLGNSAYAS	HFNKVGKNVD	KWLWMLGVHR	VMSRGEGDCD	VVKSKHGSIE	ANFRAWKTKF	ISQLQALQKG	ERKKSCGGHC	KKGKCESHQH	GSEEREEGSQ	EQDELHHRDT
250	260	270	280	290	300	310	320	330	340	350	360
KEEEPFESSS	EEEFGGEDHQ	SLNSIVDVED	LGKIMDHVKK	EKREKEQQEE	KSGLFRNMGR	NEDGERAMI	TPALREALTK	QVDAPRERSL	LQTHILWNES	HRCMETTPSL	ACANKCVFCW
370	380	390	400	410	420	430	440	450	460	470	480
WHHNNPVGTE	WLWKMDQPEM	ILKEAIENHQ	NMIKQFKGVP	GVKAERFEEG	MTVKHCALSL	VGEPIMYPEI	NRFLKLLHQC	KISSFLVTNA	QFPAEIRNLE	PVTQLYVSVD	ASTKDSLKKI
490	500	510	520	530	540	550	560	570	580	590	600
DRPLFKDFWQ	QFLDSLKALA	VKQQRTVYRL	MLVKAWNVDE	LQAYAQLVSL	GNPDFIEVKG	VTYCRESSAS	SLTMAHVPWH	EEVVQFVREL	VDLIPEYEIA	CEHEHSNCLL	IAHRKFKIGG
610	620	630	640	650	660	670					
EWWTWIDYNR	FQELIQEYED	SGGSKTFSAK	DYMARTPHWA	LFGANERSFD	PKDTRHQRKN	KSKAISGC					

Cmpd.	No. of Cmpds.	m/z meas.	Δ m/z [ppm]	z	Rt [min]	Score	Site [%]	P	Range	Sequence	Modification	Type
29	1	1044.5290	-66.32	1	15.55	36.05		1	307-315	R.RAMITPALR.E	Oxidation: 3	CID

Protein Report

**Protein 26:**	Chain A, Solution Structure Of The Conserved C-Terminal Dimerization Domain Of Borealin		
**Accession:**	gi|242556145	**Score:**	34.82
**Database:**	NCBInr	**MW [kDa]:**	8.10
**Seq. Coverage [%]:**	19.70 %	**pI:**	9.99
		**No. of Peptides:**	1

```
         10         20         30         40         50         60         70         80
GSAGERIYNI SGNGSPLADS KEIFLTVPVG GGESLRLLAS DLQRHSIAQL DPEALGNIKK LSNRLAQICS SIRTHK
```

Cmpd.	No. of Cmpds.	m/z meas.	Δ m/z [ppm]	z	Rt [min]	Score	Site [%]	P	Range	Sequence	Modification	Type
36	1	1605.8640	2.78	1	15.25	34.82		0	45-59	R.HSIAQLDPEALGNIK.K		CID

Table S2: Densitometry assessments for Western blot from figure 3A.

Relative Protein Amounts Cell Lines			
	**CDCA8**	Actin	relative density
OVCAR3	10550.7	28657.71	0.368162761
OVCAR4	9258.459	27857.42	0.332351656
CAOV3	10755.12	26297.83	0.408973485
LP9	30.823	27848	0.00110683
	**FNDC3B**	Actin	relative density
OVCAR3	38829.69	28657.71	1.354947469
OVCAR4	40168.94	27857.42	1.441947563
CAOV3	39380.64	26297.83	1.497486295
LP9	39891.94	27848	1.43248816
	**KRT14**	Actin	relative density
OVCAR3	17086.87	28657.71	0.596240014
OVCAR4	16536.63	27857.42	0.593616788
CAOV3	17339.58	26297.83	0.659354011
LP9	54.903	27848	0.001971524
	**HNRN**	Actin	relative density
OVCAR3	19246.28	28657.71	0.671591635
OVCAR4	18884.45	27857.42	0.677896674
CAOV3	19791.4	26297.83	0.752586791
LP9	52.108	27848	0.001871157

Table S3: Western Blot Densitometry

	K14	actin	relative density	reduction
UT	4661.569	35919.26	0.129779101	1
NT	4208.861	36190.67	0.116296843	0.896113796
gs1	764.82	36706.79	0.020835925	0.160549154
gs2	506.799	37027.26	0.013687187	0.105465262
gs3	5567.933	36776.11	0.151400823	1.166604035

	cdca8	actin	relative density	reduction
NT	15806.21	32181.73	0.491154686	1
gs1	2245.719	33138.97	0.067766703	0.137974257
gs2	23505.47	33924.44	0.69287725	1.41071086
gs3	18845.23	32780.68	0.57488827	1.170483121

	HNRN	actin	relative density	reduction
UT	11245.81	11013.34	1.021108038	1
NT	10797.92	12764.22	0.845952713	0.828465433
gs1	846.648	11741.75	0.072105764	0.070615215
gs2	297.778	4245.548	0.070138884	0.068688994
gs3	846.648	12130.02	0.069797718	0.068354881

Figure S1: Western Blot screening following CRISPR transfection and selection.

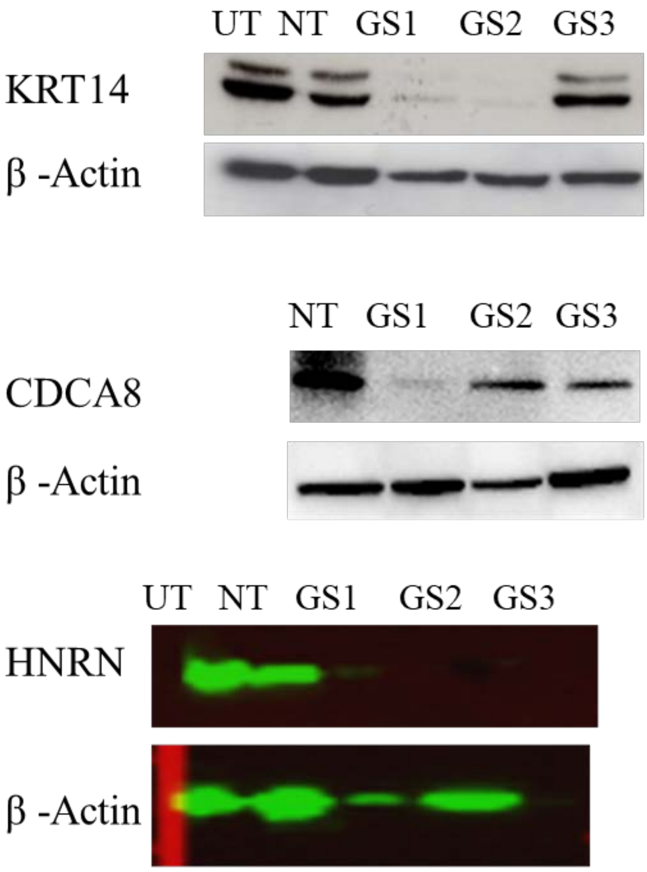

Table S4: Patient sample details for TMA analyses

Variables	
Total patient number	292
Age	12 to 76
Median	46
FIGO Grade and Stage	
I-I	15
I-Ia	9
I-Ib	3
I-Ic	1
I-II	2
I-IIa	3
I-IIb	2
I-IIIb	1
I-IIIc	1
I-IV	1
II-I	8
II-IA	10
II-IB	4
II-IC	3
II-II	6
II-IIA	2
II-IIC	1
II-IIIC	10
II-IV	4
III-I	10
III-IA	20
III-IB	13
III-IC	6
III-II	12
III-IIA	10
III-IIB	4
III-III	1
III-IIIc	12
III-IV	6
Normal/Benign	18
Ungraded/Unstaged	94
Histological Subtype	
Low Grade Serous	27
High Grade Serous	54
Papillary	44
Adenocarcinoma	4
Mucinous	14
Endometrioid	14
Clear cells	19
Normal	10
Fallopian Tube	8
GCT	34
Mixed	64

Figure S2: KRT14 and H&E staining of USBiomax TMAs

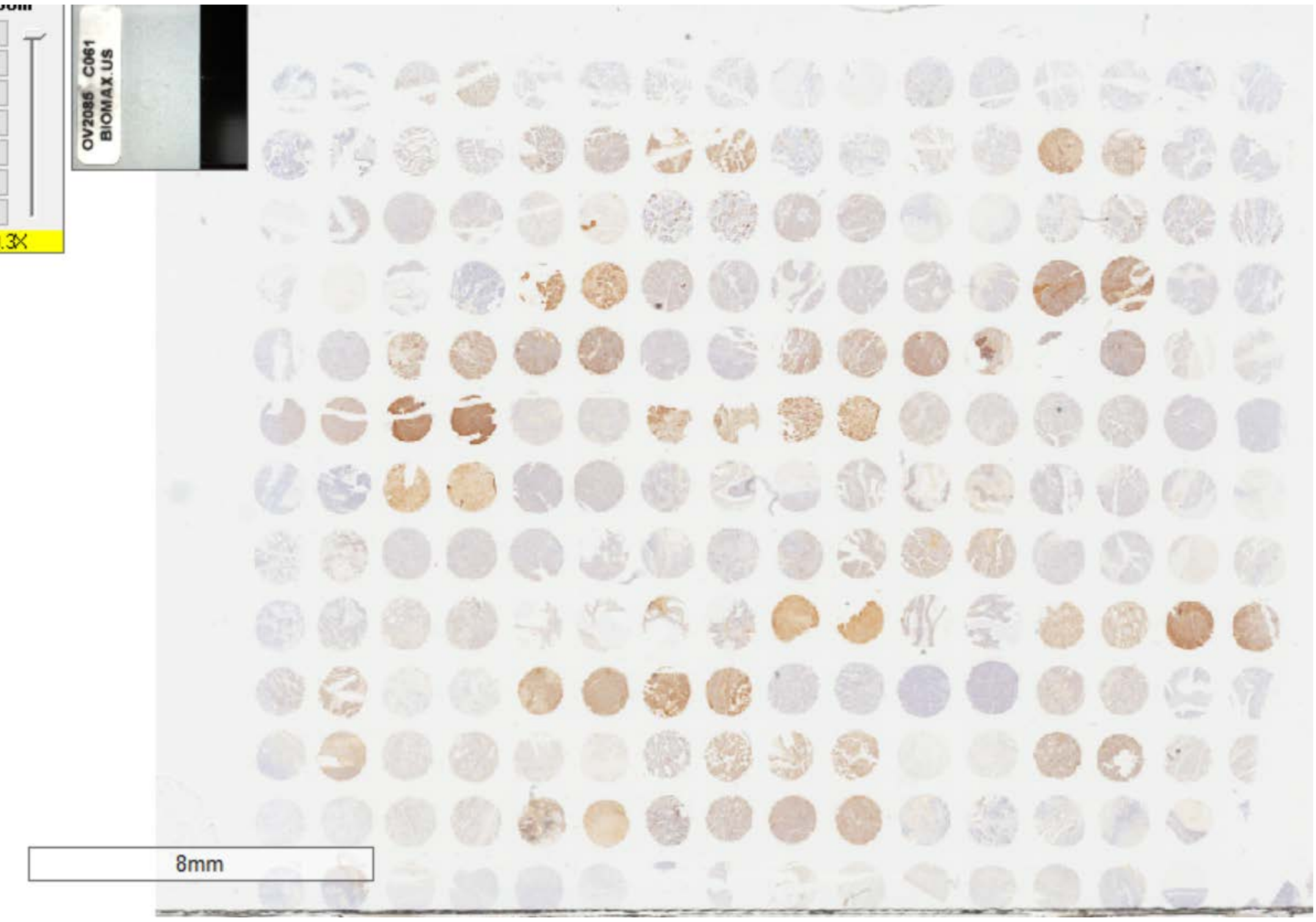

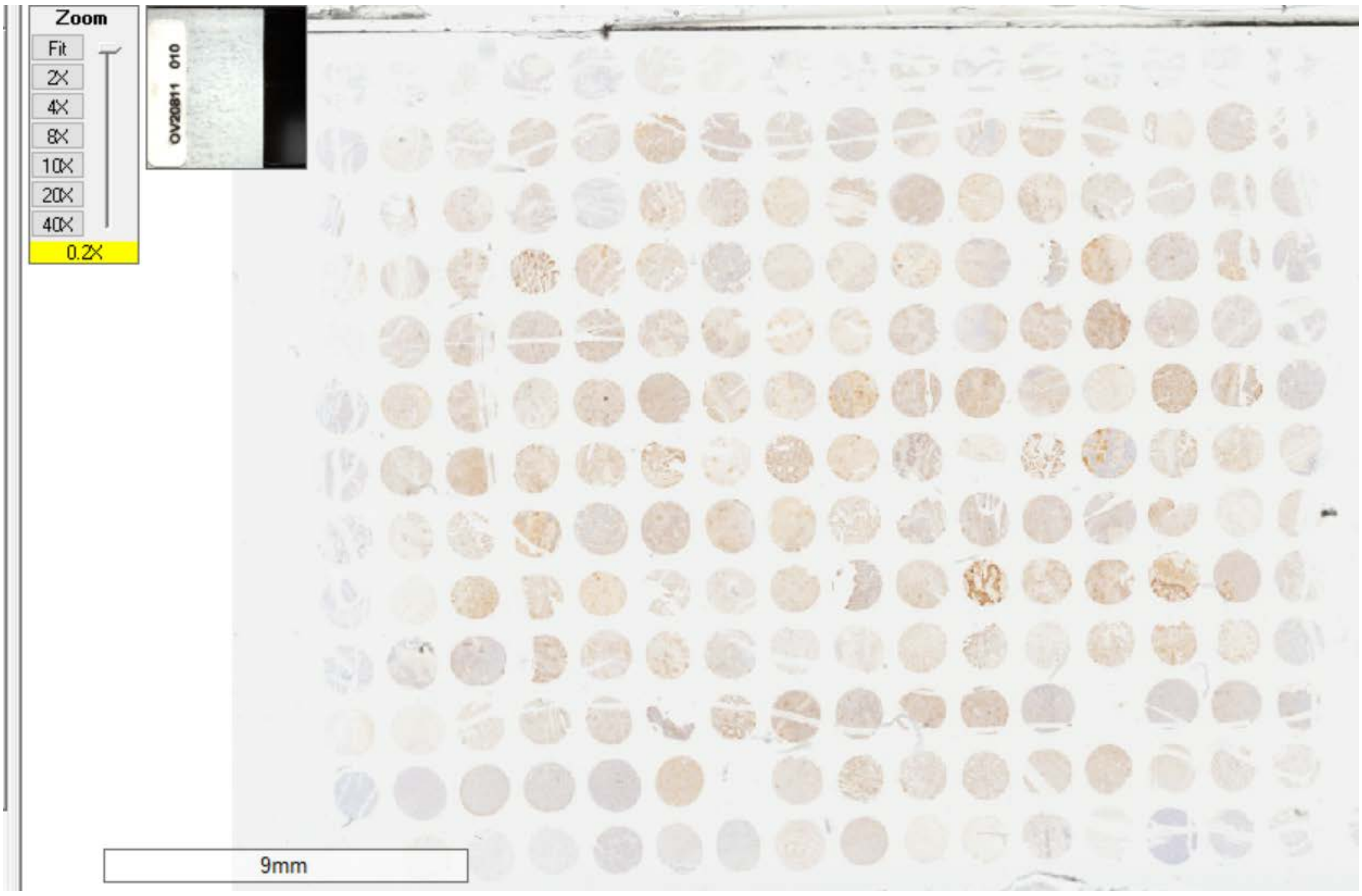

Figure S3: KRT14 antibody specificity on wild type, overexpressing and K14 knockout cells – OVCAR4

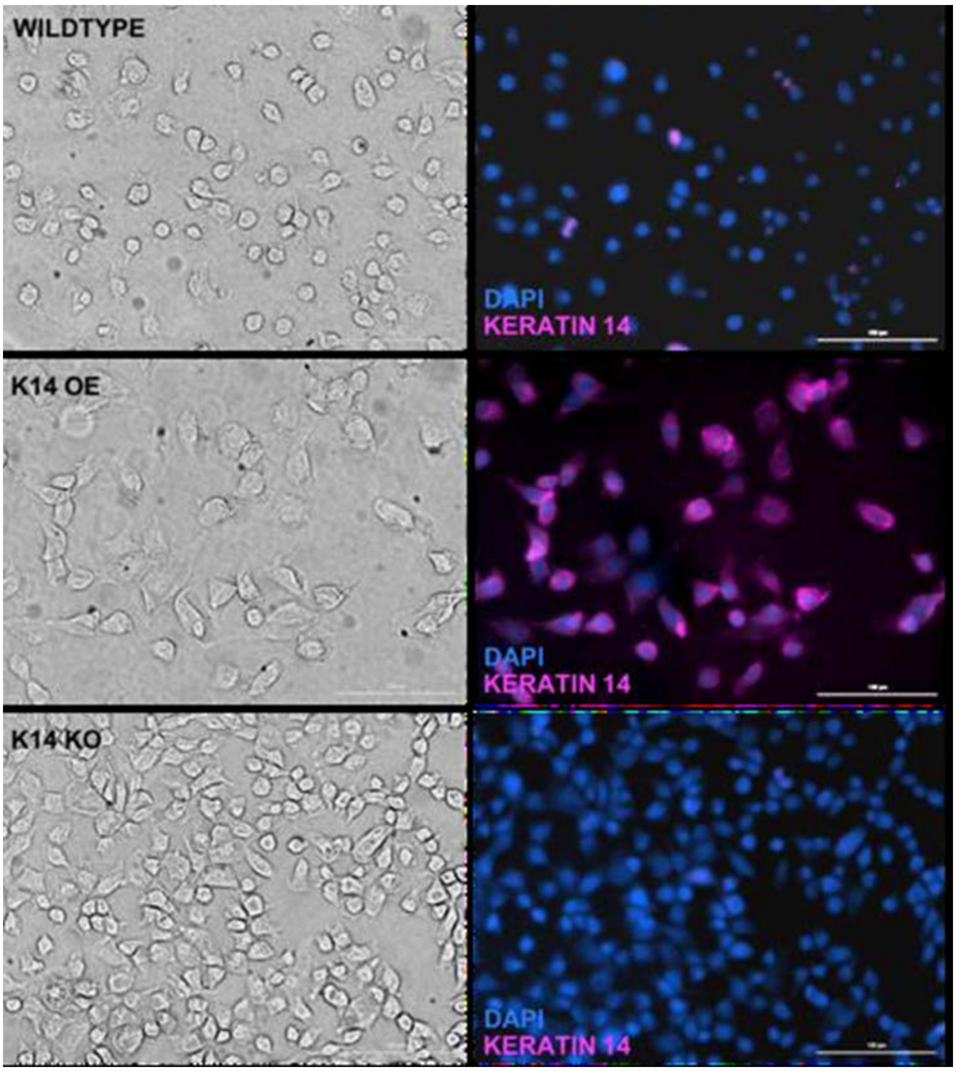

Figure S4: xCELLigence Proliferation and invasion - CaOV3

PROLIFERATION

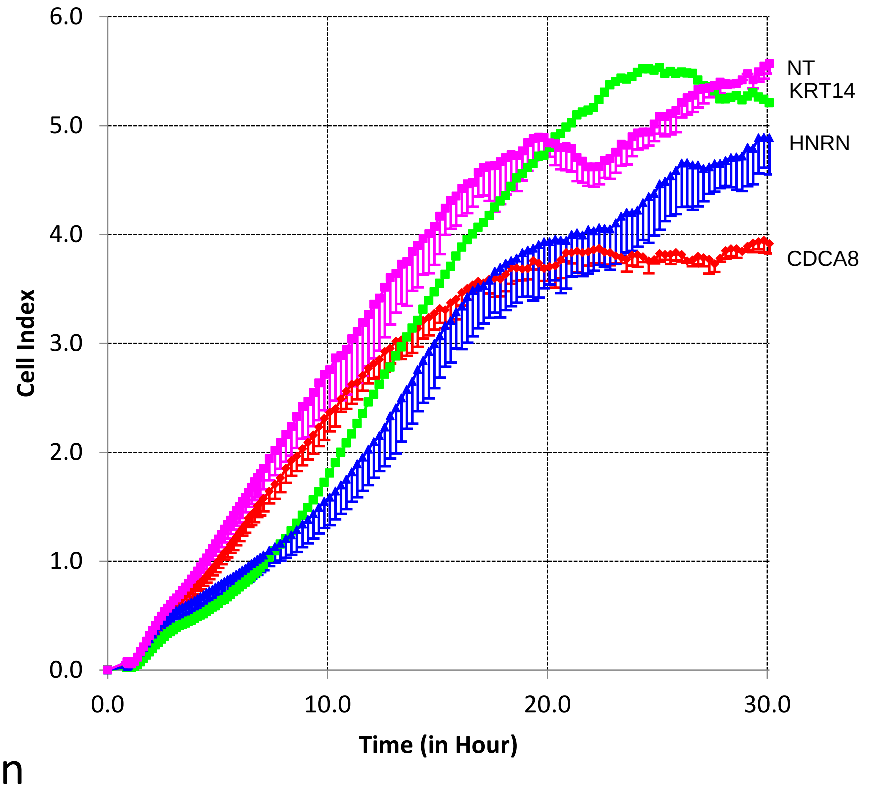

Invasion

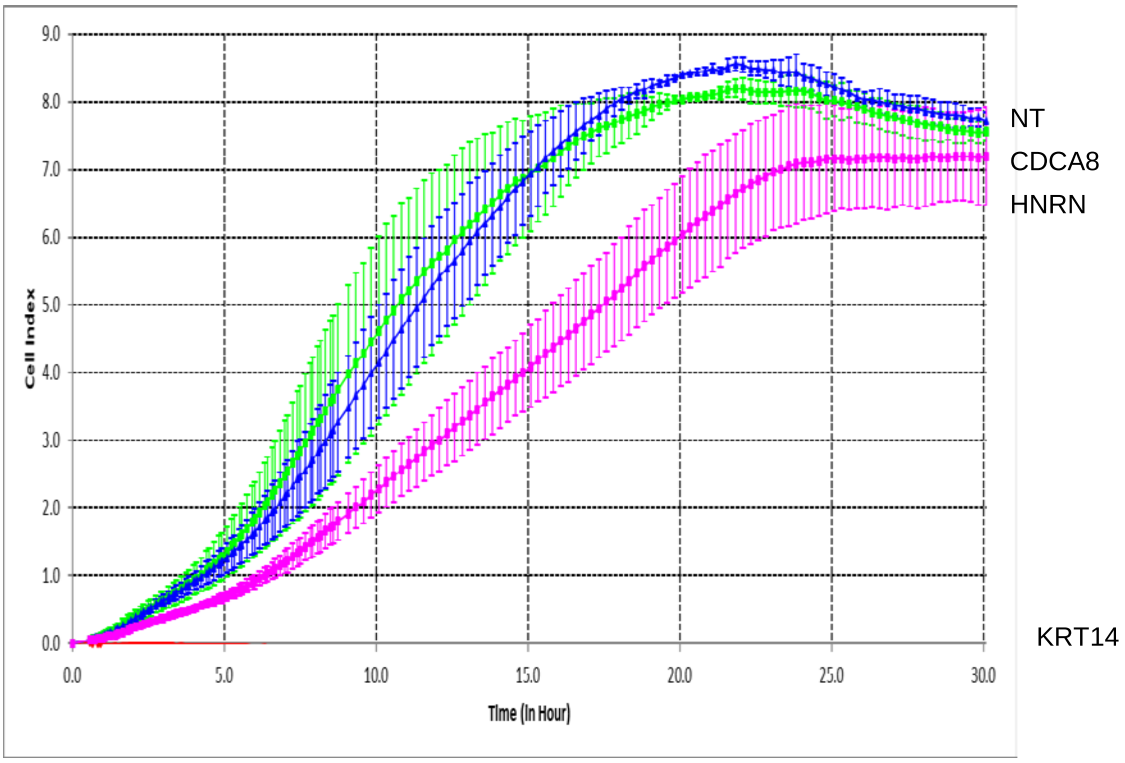

Table S5: CRISPR sgRNA sequences to target candidates.

**Gene name**	**Exon number**	**sgRNA sequence (25 mer)**
HRNR	1	ACGTATCATACTCCCCATGC
HRNR	1	GGAGTATGATACGTTGAACA
HRNR	2	GCGGCCCGAAGCGTGATGGG
HRNR	2	GTCGGACACGGCTAGGAGAG
HRNR	2	TTTGGTCGATCTTCCAGCAG
CDCA8	1	CTGAAAGACTTCGACCGTGA
CDCA8	3	TTACTGTTGCCGCCTCTTCC
CDCA8	3	AGCCCTTGGAGGAAACAAAC
KRT14	1	TCATCCTCCCGCTTCTCCTC
KRT14	1	TGGTGGCCTTGGTGCTGGCT
KRT14	2	GCAGATTCTCACAGCCACAG
FNDC3B	1	GATGACCGACCAAATCCCTC
FNDC3B	2	GCTACCTCTCCGTTCAGCAA
FNDC3B	2	ATGATGCCCCACTTGGTGAA
FNDC3B	2	GACTTTCACAATAAGAGCAG
FNDC3B	2	GGAACACTTCAGTGCATTCA
